# TRPV4 disrupts mitochondrial transport and causes axonal degeneration via a CaMKII-dependent elevation of intracellular Ca^2+^

**DOI:** 10.1038/s41467-020-16411-5

**Published:** 2020-05-29

**Authors:** Brian M. Woolums, Brett A. McCray, Hyun Sung, Masashi Tabuchi, Jeremy M. Sullivan, Kendra Takle Ruppell, Yunpeng Yang, Catherine Mamah, William H. Aisenberg, Pamela C. Saavedra-Rivera, Bryan S. Larin, Alexander R. Lau, Douglas N. Robinson, Yang Xiang, Mark N. Wu, Charlotte J. Sumner, Thomas E. Lloyd

**Affiliations:** 1 Department of Pharmacology and Molecular Sciences, Johns Hopkins University School of Medicine, Baltimore, MD USA; 2 Department of Neurology, Johns Hopkins University School of Medicine, Baltimore, MD USA; 3 Neurobiology Department, UMass Medical School, Worcester, MA USA; 4 Department of Cell Biology, Johns Hopkins University School of Medicine, Baltimore, MD USA; 5 The Solomon H. Snyder Department of Neuroscience, Johns Hopkins University School of Medicine, Baltimore, MD USA

**Keywords:** Cellular neuroscience, Genetics of the nervous system, Ion channels in the nervous system, Somatic system, Peripheral neuropathies

## Abstract

The cation channel transient receptor potential vanilloid 4 (TRPV4) is one of the few identified ion channels that can directly cause inherited neurodegeneration syndromes, but the molecular mechanisms are unknown. Here, we show that in vivo expression of a neuropathy-causing TRPV4 mutant (TRPV4^R269C^) causes dose-dependent neuronal dysfunction and axonal degeneration, which are rescued by genetic or pharmacological blockade of TRPV4 channel activity. TRPV4^R269C^ triggers increased intracellular Ca^2+^ through a Ca^2+^/calmodulin-dependent protein kinase II (CaMKII)-mediated mechanism, and CaMKII inhibition prevents both increased intracellular Ca^2+^ and neurotoxicity in *Drosophila* and cultured primary mouse neurons. Importantly, TRPV4 activity impairs axonal mitochondrial transport, and TRPV4-mediated neurotoxicity is modulated by the Ca^2+^-binding mitochondrial GTPase Miro. Our data highlight an integral role for CaMKII in neuronal TRPV4-associated Ca^2+^ responses, the importance of tightly regulated Ca^2+^ dynamics for mitochondrial axonal transport, and the therapeutic promise of TRPV4 antagonists for patients with TRPV4-related neurodegenerative diseases.

## Abstract (web summary)

Mutations in the TRPV4 channel cause inherited neurodegeneration syndromes, but the molecular mechanisms are unknown. Here the authors reveal that TRPV4 activation causes dose-dependent, CaMKII-mediated neuronal dysfunction and axonal degeneration via disruption of mitochondrial axonal transport.

## Introduction

Congenital distal spinal muscular atrophy (CDSMA), scapuloperoneal spinal muscular atrophy (SPSMA), and Charcot-Marie-Tooth disease type 2C (CMT2C) are inherited degenerative diseases of the peripheral nervous system caused by mutations in the gene encoding the transient receptor potential vanilloid 4 (TRPV4) cation channel^1–3^. Peripheral nerve degeneration in these disorders results in muscle weakness, particularly of limb, diaphragm, and vocal fold muscles, the latter of which can be life threatening. They are strikingly variable in severity, ranging from severe, congenital onset to mild, late adult onset. The mutations are dominantly inherited missense mutations and each is capable of causing a wide spectrum of disease severities even within the same family.

TRPV4 is a cell surface-expressed, non-selective cation channel that is preferentially permeable to Ca^2+^ and is activated by mechanical, osmotic, and chemical stimuli^4^. The majority of neuropathogenic mutations are present in the intracellular amino-terminal ankyrin repeat domain^1–3,5,6^, where it has been postulated that they may alter inter- or intra-protein–protein interactions. The consequences of mutations of *TRPV4* have been studied in cultured cells with conflicting results. Some studies suggest that neuropathy-causing mutations lead to a gain of TRPV4 ion channel function^1,2,5,6^, whereas others argue they cause a loss of function^3^. No studies have yet examined the effects of neuropathy-causing *TRPV4* mutations on neurons in vivo. Establishing the pathogenic mechanisms of *TRPV4* mutations has particular relevance for therapeutics development, as small molecule TRPV4 antagonists have proven safe in human clinical trials^7^ and could be repurposed for neurological disease indications.

There are very few examples of ion channels that are directly implicated in the process of neurodegeneration, as most neurological disease-associated channelopathies are paroxysmal disorders such as epilepsy or migraine^8^. Investigating how *TRPV4* mutations cause peripheral neuropathy provides an opportunity to understand the molecular events linking an ion channel and Ca^2+^ homeostasis to the process of neurodegeneration. Although Ca^2+^ homeostasis is dysregulated in many neurodegenerative disorders, it is unknown whether Ca^2+^ dysregulation is a primary or secondary pathological event.

Ca^2+^ regulates both the initiation of fast axonal transport as well as sustained transport of cargos along axons^9,10^, and disruptions of axonal transport are implicated in many neurodegenerative diseases, particularly peripheral nerve disease^11,12^. Several forms of hereditary neuropathy are caused by mutations in genes encoding proteins that regulate axonal transport such as kinesin (*KIF5A*), dynein (*DYNC1H1*), and neurofilament (*NEFL*)^12^. Moreover, the most common form of axonal CMT is caused by mutations of mitofusin 2 (*MFN2*), which are associated with impaired axonal transport of mitochondria^13–15^. Interrogation of the cellular events underlying TRPV4-associated neuropathy may provide insights into mechanistic links between Ca^2+^ and impaired axonal transport in neurodegenerative disease.

In this study, we explored the consequences of mutant TRPV4 expression in *Drosophila* and cultured primary mammalian neurons. We show that mutant TRPV4 causes neuronal dysfunction and degeneration that are dependent on TRPV4 channel activity. Using an unbiased forward genetic screen in *Drosophila*, we found that TRPV4-mediated increases in intracellular Ca^2+^ require CaMKII, revealing a central role for CaMKII in TRPV4-associated axonal degeneration. Moreover, we observe axonal mitochondrial transport defects downstream of TRPV4 channel activation. Our data suggest that neuropathogenic TRPV4 mutations sensitize the TRPV4 ion channel resulting in CaMKII-dependent Ca^2+^ entry that both disrupts mitochondrial axon transport and causes axonal degeneration.

## Results

### TRPV4 mutations disrupt neuron function via the TRPV4 pore

**Fig. 1:**
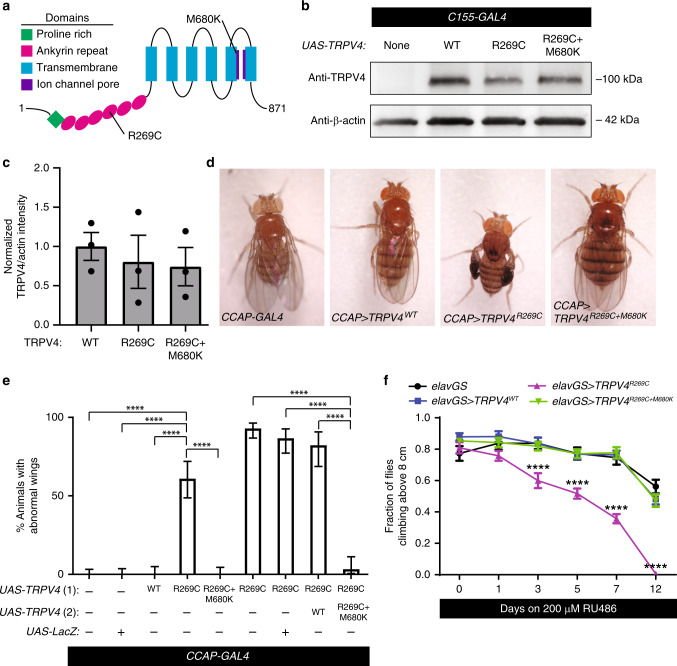
A neuropathy-causing TRPV4 variant causes channel pore-dependent neuronal dysfunction in *Drosophila*. **a** Schematic of TRPV4 domain structure with neuropathy-causing (R269C) and pore-inactivating (M680K) mutations indicated. **b** Representative western blot of protein lysates from the heads of adult *Drosophila* expressing TRPV4(mod) variants under the control of *C155*-*GAL4*. **c** Normalized mean ± SEM band intensities from three independent western blots. One-way ANOVA (*p* = 0.778). **d** Images of flies expressing no TRPV4, TRPV4^WT^(mod), TRPV4^R269C^(mod), and TRPV4^R269C+M680K^(mod) in N_CCAP_. **e** Percentage ± 95% CI of flies with unexpanded wings in **d**. From left to right *n* = 115, 102, 73, 64, 82, 114, 75, 45, and 61 flies. *Χ*^2^ of all groups (*p* < 0.0001) followed by pairwise two-sided Fisher’s exact test. **f** Climbing performance of flies inducibly expressing TRPV4 (high) variants. Flies induced at 1–3 days post eclosion with 200 μM RU486. Mean ± SEM. *n* = 10 vials of 10 flies per genotype. Two-way ANOVA (*p* < 0.0001), Tukey’s post hoc test, asterisks indicate difference from all other genotypes. For all panels: ****p < 0.0001.

Human TRPV channel expression rescues phenotypes resulting from loss-of-function mutations in *Drosophila* TRPV channels, demonstrating functional conservation across species^16^. To evaluate neuropathogenic *TRPV4* mutations in vivo, we generated transgenic *Drosophila* lines that express human TRPV4 under the control of the *GAL4/UAS* binary expression system. We primarily utilized three TRPV4 variants in our studies: wild type TRPV4 (TRPV4^WT^), a neuropathy-causing mutant (TRPV4^R269C^), and TRPV4^R269C^ with a second engineered mutation known to block the TRPV4 ion-conducting pore (TRPV4^R269C+M680K^) (Fig. 1a)^1^. We identified low-, moderate-, and high-expressing transgenic lines (TRPV4(low), TRPV4(mod), and TRPV4(high)) in which these three variants are expressed at similar levels (Fig. 1b, c, Supplementary Fig. 1a, b). When expressed in all neurons using the *C155-GAL4* driver, flies expressing TRPV4^R269C^, but not TRPV4^WT^ or TRPV4^R269C+M680K^, fail to appropriately expand their wings after eclosion (Supplementary Fig. 1c). This phenotype is dose-dependent, as high-level expression of TRPV4^R269C^ markedly increases the penetrance of the wing phenotype (Supplementary Fig. 1c). A second neuropathy-causing mutant (TRPV4^R232C^) also causes this wing expansion phenotype (Supplementary Fig. 1c), suggesting that this phenotype is common to neuropathy-associated variants.

*Drosophila* wing expansion is controlled by crustacean cardioactive peptide-expressing neurons (N_CCAP_), which initiate motor programs upon eclosion that drive wing expansion^17,18^. Selective expression of TRPV4^R269C^(mod) in these neurons using *CCAP-GAL4* recapitulates the unexpanded wing phenotype observed with pan-neuronal expression (Fig. 1d, e). Flies expressing TRPV4^R269C+M680K^ have no wing phenotype, even with high-level pan-neuronal expression (Fig. 1d, e, Supplementary Fig. 1c). Furthermore, co-expression of TRPV4^R269C+M680K^(mod) strongly suppresses the phenotype caused by TRPV4^R269C^(mod), suggesting that the pore-inactivating mutation blocks channel function both in *cis* and in *trans* (Fig. 1e), consistent with the known tetrameric structure of TRPV4 ion channels^19^.

*TRPV4* mutations are associated with congenital onset disease in humans, but also with later onset, slowly progressive symptoms. To assess whether mutant TRPV4 can cause progressive disease after adult development, we utilized an inducible pan-neuronal GAL4 driver *elav*-*GeneSwitch* (*elavGS*)^20^ to express TRPV4(high) variants in early adulthood. Flies induced to express TRPV4^R269C^(high) by feeding with RU486 show a marked, progressive decline in climbing performance and are unable to climb 12 days after induction of expression (Fig. 1f). In contrast, flies expressing either TRPV4^WT^(high) or TRPV4^R269C+M680K^(high) show no difference in climbing performance compared with flies carrying *elavGS* alone (Fig. 1f). Together, these data demonstrate that TRPV4^R269C^ expression can cause both early- and late-onset neuronal dysfunction in vivo, and that this neurotoxicity requires a functional ion channel pore.

### TRPV4^R269C^ causes axonal and dendritic degeneration

**Fig. 2:**
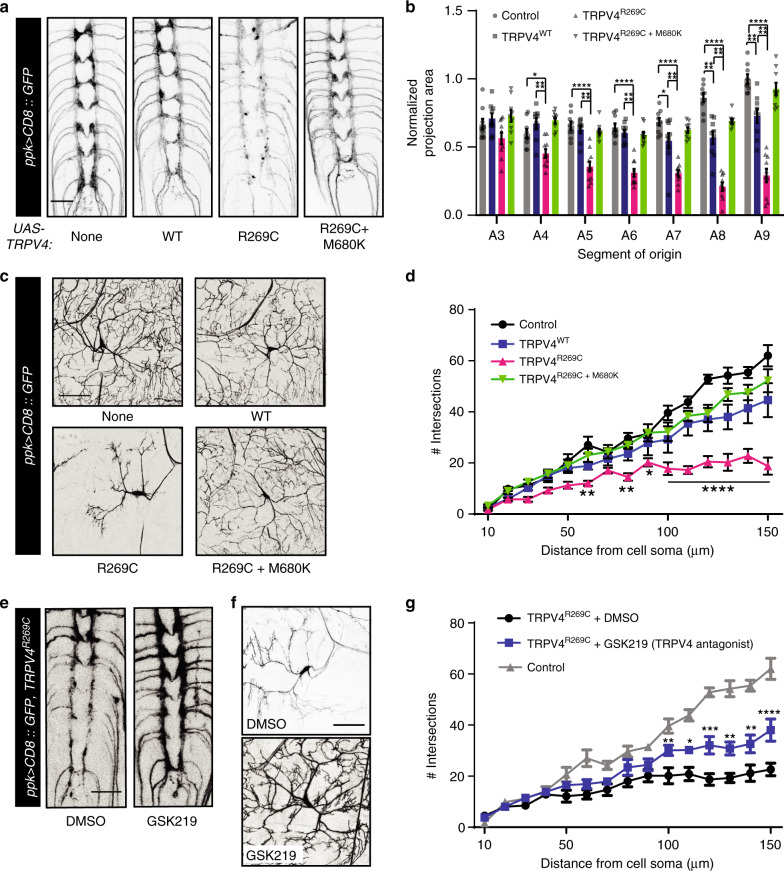
High TRPV4^R^^269C^ expression disrupts C4da neuron axonal and dendritic projections. Confocal projections of C4da neuron axonal projections **a** and dendrites **c** from wandering third instar larvae expressing TRPV4 (high) variants. **b** Mean ± SEM normalized projection areas in **a**. *n* = 9 (control), 9 (TRPV4^WT^), 10 (TRPV4^R269C^), and 9 (TRPV4^R269C+M680K^) larvae. Two-way ANOVA (*p* < 0.0001), Tukey’s post hoc test. **d** Sholl analysis of neurons in **c**. Mean ± SEM. *n* = 5 cells, one per larva, from five larvae per genotype. Asterisks denote difference from no TRPV4 (control). Two-way ANOVA (*p* < 0.0001), Tukey’s post hoc test. Confocal stacks of axonal projections **e** and dendrites **f** of flies expressing TRPV4^R269C^(high) raised on food with either DMSO or 100 μM GSK219. **g** Sholl analysis of dendritic phenotypes in **f**. Control from **d** shown for reference. Mean ± SEM. *n* = 6 cells from three larvae for DMSO, *n* = 10 cells from five larvae for GSK219. Asterisks denote differences from DMSO. Two-way ANOVA (*p* < 0.0001), Tukey’s post hoc test. Scale bar, 25 µm in **a** and **e** and 50 µm in **c** and **f**. For all panels: *p < 0.05, **p < 0.01, ***p < 0.001, and ****p < 0.0001.

Degeneration and loss of peripheral nerve axons are characteristic pathological features of CMT^21^. To test whether TRPV4^R269C^ causes neuronal degeneration, we assessed class IV larval dendritic arborization (C4da) neurons, sensory neurons with dendrites that tile the *Drosophila* larval body wall^22^, in wandering third instar larvae using *ppk-GAL4*^23^. We selected these neurons for functional and morphological analysis owing to their genetic tractability, experimental accessibility, and their previous use in a *Drosophila* model of CMT^24^. As compared with control flies expressing TRPV4^WT^(high), TRPV4^R269C+M680K^(high), or no TRPV4, flies expressing TRPV4^R269C^(high) show a marked loss of C4da neuron axonal projections into the ventral nerve cord (Fig. 2a, b) and severely reduced dendritic arborizations within the body wall (Fig. 2c, d). These phenotypes are not observed with overexpression of other cation channels previously shown to activate or silence *Drosophila* neurons and disrupt N_CCAP_ function^18,25^ (Supplementary Fig. 2a, c), suggesting these axonal and dendritic degeneration phenotypes are not caused by altered neuronal activity.

To determine whether the morphological changes in C4da neurons are owing to degeneration or impaired development, we imaged C4da neuron axonal projections and dendrites in larvae at 96 hours (late second instar) and 120 hours (early third instar) after egg laying (AEL). At 96 hours AEL, we observe no significant differences in either axonal projections or number of dendritic branches (Supplementary Fig. 3a, c). However, at 120 hours AEL, we observe axonal swellings (Supplementary Fig. 3a, 2× zoom, green arrows), fragmentation of distal axonal projections (Supplementary Fig. 3a, 2× zoom, magenta arrow), and a reduction of dendritic branching (Supplementary Fig. 3b, c) in larvae expressing TRPV4^R269C^ (high). Like the phenotypes in N_CCAP_, axonal degeneration in C4da neurons is TRPV4-dosage dependent, as moderate expression of TRPV4^R269C^ does not alter axonal projection area (Supplementary Fig. 3d, e). Of note, high expression (but not moderate expression) of TRPV4^WT^ causes a mild wing expansion phenotype and a mild loss of axonal projections and dendritic branching (Supplementary Fig. 1c, Fig. 2a, b). Thus, TRPV4^R269C^ causes neurodegeneration in vivo in a dose-dependent manner, and sufficient expression of TRPV4^WT^ can cause similar, yet more mild, phenotypes. These observations are consistent with a gain-of-TRPV4 function mechanism of toxicity caused by the R269C mutation.

### TRPV4 antagonists suppress TRPV4-mediated neurodegeneration

As genetic inactivation of the TRPV4^R269C^ channel pore rescues neurotoxicity (Figs. 1d, f, 2a, d, and Supplementary Fig. 1c), we next asked if TRPV4 could be blocked pharmacologically to suppress the axonal and dendritic phenotypes mediated by TRPV4^R269C^(high). We tested the selective TRPV4 antagonist GSK2193874 (GSK219) because TRPV4 antagonists are in clinical development for the treatment of pulmonary edema during heart failure^7,26^. Embryos expressing TRPV4^R269C^ were raised on food containing either GSK219 or DMSO vehicle alone throughout larval development. Remarkably, GSK219 treatment of TRPV4^R269C^(high) flies reduces axonal degeneration and dendritic branching defects as compared to vehicle treatment alone (Fig. 2e, g).

### CaMKII is required for TRPV4^R269C^-mediated neurotoxicity

**Fig. 3:**
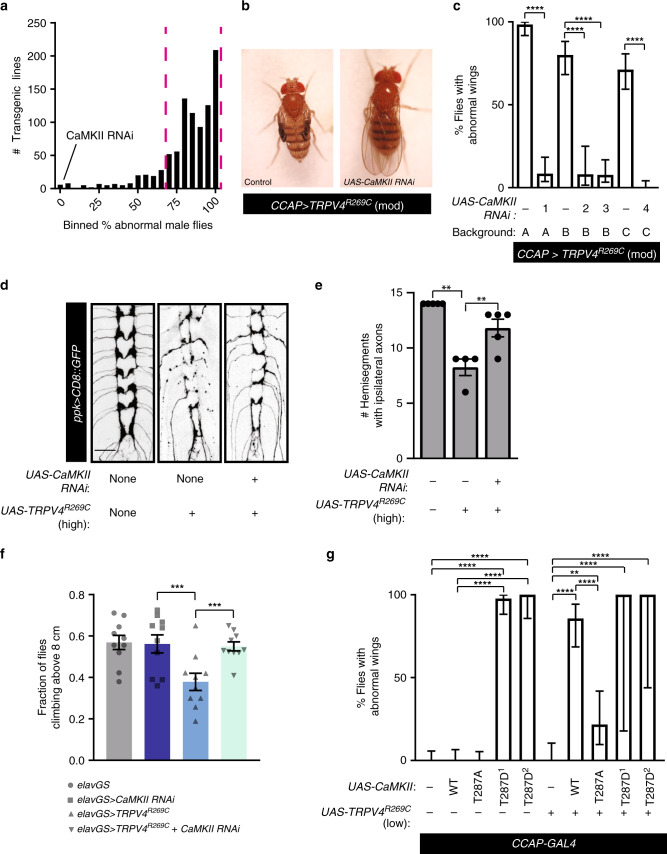
CaMKII is required for TRPV4^R269C^-mediated neuronal toxicity. **a** Histogram of results from genetic modifier screen against TRPV4^R269C^ toxicity in N_CCAP_. Dashed magenta lines denote upper and lower bounds of control lines. **b** Images of flies expressing TRPV4^R269C^(mod) ± CaMKII RNAi. **c** Percentage ± 95% CI of flies with unexpanded wings when co-expressing TRPV4^R269C^(mod) and different CaMKII RNAi fly lines. A = TRiP collection lines, B = Vienna GD collection lines, and C = Vienna KK collection lines. From left to right *n* = 65, 59, 60, 25, 65, 66, and 90 flies. *Χ*^2^ test of all groups (*p* < 0.0001) followed by pairwise two-sided Fisher’s exact test. **d** Confocal stacks of axonal projections in C4da neurons expressing TRPV4^R269C^(high) ± CaMKII RNAi. **e** Mean ± SEM innervation in **d**. *n* = 5, 4, and 5 larvae, respectively. One-way ANOVA (*p* < 0.0002), Tukey’s post hoc test. Scale bar, 25 µm. **f** Climbing performance of flies 7 days after induction of expression of TRPV4^R269C^(high) ± CaMKII RNAi with 200 μM RU486. Mean ± SEM. *n* = 10 vials of 10 flies per genotype. Two-way ANOVA (*p* < 0.0001), Tukey’s post hoc test. **g** Percentage ± 95% CI of flies with unexpanded wings when co-expressing TRPV4^R269C^(low) with variants of CaMKII. T287D^1^ and T287D^2^ denote independent *UAS*-*CaMKII*^*T287D*^ lines. From left to right *n* = 63, 55, 68, 44, 23, 33, 28, 23, 2, and 3 flies. *Χ*^2^ test of all groups (*p* < 0.0001) followed by pairwise two-sided Fisher’s exact test. For all panels: **p < 0.01, ****p < 0.0001.

To identify genes that contribute to TRPV4^R269C^-mediated neurotoxicity, we performed RNAi- and overexpression-based screens for genetic modifiers of the wing expansion phenotype caused by moderate TRPV4^R269C^ expression in N_CCAP_. With the goal of identifying potential therapeutic targets, we specifically screened genes that are conserved in humans, potentially druggable^27^, and expressed in the *Drosophila* nervous system (flybase.org). We also screened fly orthologues of genes previously implicated in CMT. In total, we evaluated 692 transgenic lines covering 502 genes and identified 8 genes that reduce the penetrance of the TRPV4^R269C^-mediated wing phenotype to <25% compared with the 70–100% penetrance observed in control flies (Fig. 3a, Supplementary Data 1).

The most potent genetic modifier identified in this screen was Ca^2+^/calmodulin-dependent protein kinase II (CaMKII), a key regulatory kinase of many neuronal signaling pathways^28^. Knockdown of the single *Drosophila* CaMKII using four independent RNAi lines on three distinct genetic backgrounds reduces the penetrance of the TRPV4^R269C^-mediated wing phenotype to 0–10% (Fig. 3a, c). CaMKII knockdown is also sufficient to ameliorate other TRPV4^R269C^-mediated phenotypes including the loss of C4da neuron axonal projections in flies expressing TRPV4^R269C^(high) (Fig. 3d, e) and the climbing phenotype at 7 days post induction of *elavGS* (Fig. 3f).

To test whether CaMKII overexpression can enhance TRPV4^R269C^-mediated neurotoxicity, we generated a *UAS* line that expresses TRPV4^R269C^ at very low levels via site-directed insertion (TRPV4^R269C^(low))^29^. Although overexpression of either TRPV4^R269C^(low) or CaMKII alone in N_CCAP_ did not impair wing expansion, simultaneous overexpression of both CaMKII and TRPV4^R269C^(low) causes a highly penetrant wing phenotype (Fig. 3g). Upon binding Ca^2+^, CaMKII is autophosphorylated at T287 enabling Ca^2+^-independent, constitutive CaMKII activity^28^. Interestingly, in the absence of TRPV4^R269C^, expression of the constitutively active T287D phosphomimetic mutant of CaMKII^30^, but not the Ca^2+^-dependent T287A mutant, cause a highly penetrant wing phenotype (Fig. 3g). These data suggest that CaMKII autophosphorylation is necessary and sufficient to mediate neurotoxicity caused by TRPV4^R269C^. A secondary genetic screen examining known CaMKII target proteins and pathways did not identify individual modifiers that similarly rescued TRPV4^R269C^-mediated toxicity, suggesting that either multiple CaMKII substrates are involved or that CaMKII is working through an unknown substrate (Supplementary Data 2).

### TRPV4^R269C^ causes CaMKII-dependent hyperexcitability

**Fig. 4:**
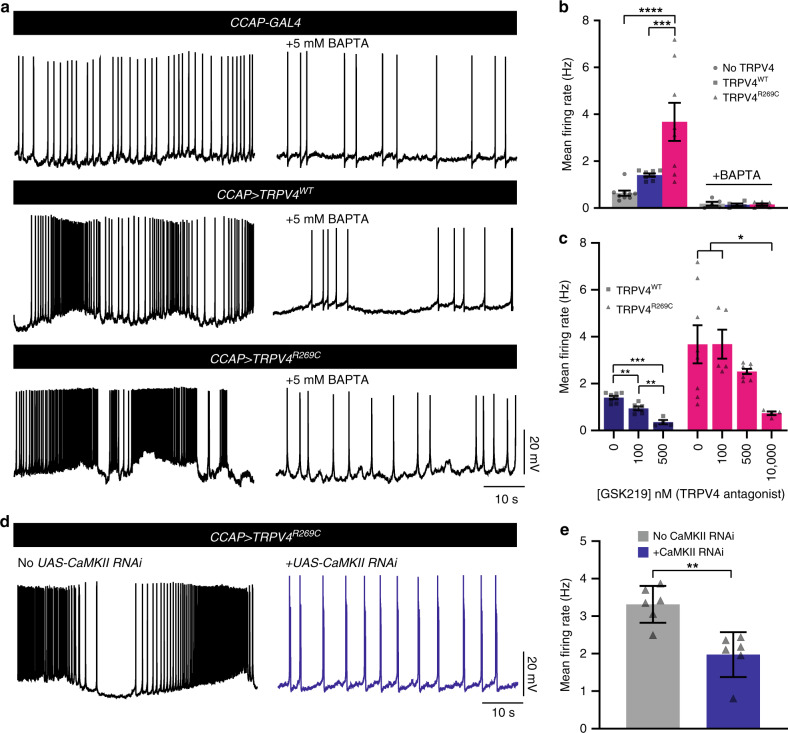
TRPV4^R269C^ mediates a reversible, Ca^2+^- and CaMKII-dependent increase in neuronal excitability. **a** Traces of N_CCAP_ spontaneous activity in flies expressing no TRPV4, TRPV4^WT^(mod), or TRPV4^R269C^(mod) ± 5 mM BAPTA. **b** Mean firing rates from **a**. Mean ± SEM. *n* = 9 (no TRPV4), 8 (TRPV4^WT^), 9 (TRPV4^R269C^), 5 (no TRPV4 + BAPTA), 5 (TRPV4^WT^ + BAPTA), and 6 (TRPV4^R269C^ + BAPTA) flies. Two-way ANOVA (*p* = 0.0017), Tukey’s post hoc test. **c** Mean firing rate after incubation with GSK219. Mean ± SEM. TRPV4^WT^ *n* = 8, 6, and 4 flies for 0, 100, and 500 nM TRPV4^R269C^ *n* = 8, 5, 8, and 4 flies for 0, 100, 500, and 10,000 nM. 0 nM values are the same as shown in **b**. One-way ANOVA (*p* < 0.0001 (TRPV4^WT^), *p* = 0.0169 (TRPV4^R269C^)), Tukey’s post hoc test. **d** Traces of N_CCAP_ spontaneous activity in flies expressing TRPV4^R269C^ ± CaMKII RNAi^.^ **e** Mean firing rate in **d**. Mean ± SEM. *n* = 6 per genotype. Unpaired two-tailed *t* test. *p* = 0.0017. For all panels: *p < 0.05, **p < 0.01, ***p < 0.001, ****p < 0.0001.

N_CCAP_ regulate *Drosophila* wing expansion in a manner that is sensitive to changes in excitability^17,18^. To test whether TRPV4 ion channel activity influences the excitability of N_CCAP_, we utilized whole-cell perforated patch-clamp recording^25,31^ to measure the activity of N_CCAP_ in flies expressing TRPV4^WT^(mod), or TRPV4^R269C^(mod). A tracer dye was injected into the neurons following recording to validate cell identity (Supplementary Fig. 4). Expression of TRPV4^R269C^(mod) increases the spontaneous mean firing rate of N_CCAP_ by 5.8-fold, compared with an approximately twofold increase brought about by TRPV4^WT^(mod) expression (Fig. 4a, b). In addition, TRPV4^R269C^(mod) increases the intrinsic excitability of N_CCAP_ as measured by the mean firing rate in response to injected current (Supplementary Fig. 5a). We observe no change in the mean resting membrane potential (RMP) (Supplementary Fig. 5b), but subthreshold membrane potential variability (Δ ramp), a property that may allow neurons to reach threshold more frequently, is increased in flies expressing TRPV4^R269C^(mod) (Supplementary Fig. 5c).

To test whether changes in excitability are related to changes in Ca^2+^, we applied the cell-permeant Ca^2+^ chelator 1,2-bis(*O*-aminophenoxy)ethane-*N,N,N’,N’*-tetraacetic acid (BAPTA). BAPTA application blocks the increased spontaneous mean firing rate (Fig. 4a, b), intrinsic N_CCAP_ excitability (Supplementary Fig. 5d) and Δ ramp (Supplementary Fig. 5f) caused by TRPV4^WT^(mod) and TRPV4^R269C^(mod). Similarly, the TRPV4-selective antagonist GSK219 also reduces spontaneous activity (Fig. 4c), intrinsic excitability (Supplementary Fig. 5g), and Δ ramp (Supplementary Fig. 5i) in a dose-dependent fashion, as does knockdown of CaMKII (Fig. 4d, e, Supplementary Fig. 6a, c). Collectively, these data demonstrate that reversible alterations of TRPV4^R269C^-mediated excitability are dependent on both intracellular Ca^2+^ and CaMKII.

### TRPV4^R269C^ has enhanced response to agonist stimulation

**Fig. 5:**
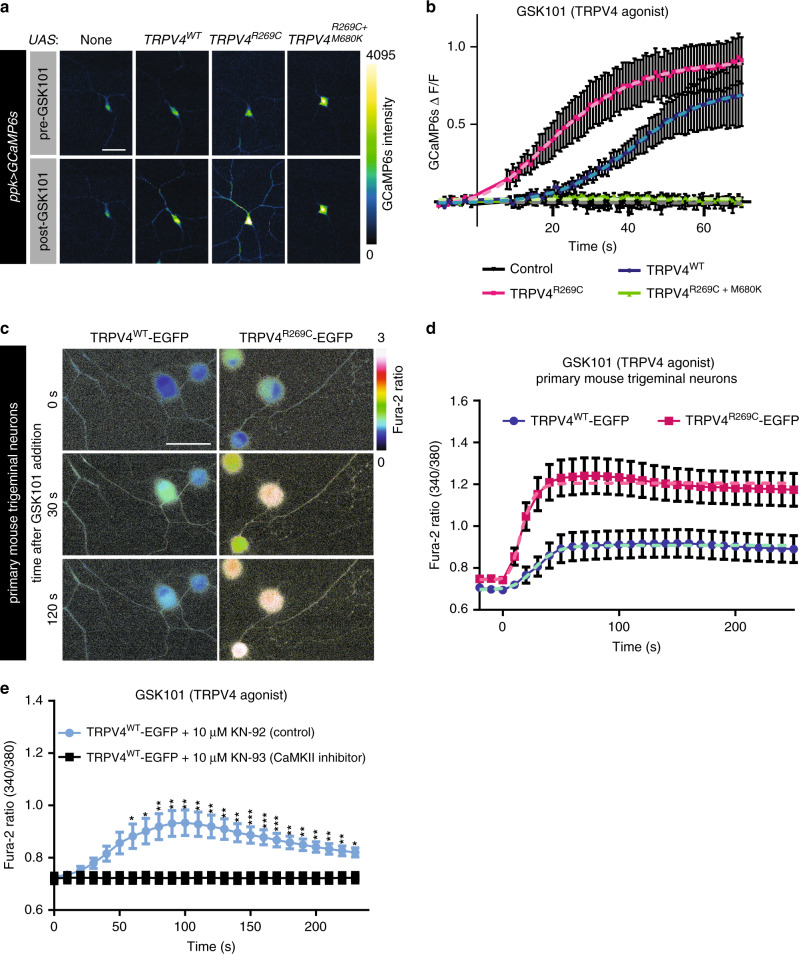
The R269C mutation enhances TRPV4-mediated Ca^2+^ influx in fly and mouse neurons. **a** Confocal images of GCaMP6s in larval C4da neurons expressing the indicated transgenes before and 180 s after application of 40 nM GSK101 at *t* = 0 s. Scale bar, 50 μm. **b** Change in somal GCaMP6s fluorescence over baseline (ΔF/F) of the genotypes shown in **a**. Mean ± SEM. *n* = 8 (no TRPV4), 8 (TRPV4^WT^), 7 (TRPV4^R269C^) and 10 (TRPV4^R269C+M680K^). Non-linear regression (dashed lines, logistic model) indicates TRPV4^WT^ *t*_1/2max_ = 41 ± 3.0 s was greater than TRPV4^R269C^ *t*_1/2max_ = 22 ± 2.4 s (*p* < 0.0001, unpaired two-tailed *t* test). Maximum response 0.91 (TRPV4^R269C^) and 0.73 (TRPV4^WT^) (*p* = 0.06, unpaired two-tailed *t* test) **c** Images of the Fura-2AM ratio in primary mouse trigeminal neurons transduced with TRPV4^WT^ or TRPV4^R269C^ before and after the application of 30 nM GSK101. Scale bar, 50 μm. **d** Mean ± SEM Fura-2AM ratio over time of the images in **c**. Drug added at *t* = 0. *n* = 44 (TRPV4^WT^) and 48 (TRPV4^R269C^) neurons from 12 wells per genotype from three separate preparations. Non-linear regression (dashed lines, logistic model) indicates TRPV4^WT^ *t*_1/2max_ = 28 ± 8.5 s and TRPV4^R269C^ *t*_1/2max_ = 16 ± 3.8 s (*p* = 0.2, unpaired two-tailed *t* test). Maximum responses TRPV4^WT^ = 0.9, TRPV4^R269C^ = 1.2 (*p* < 0.0001, unpaired two-tailed *t* test). **e** Mean ± SEM Fura-2AM ratio over time in response to 30 nM GSK101 at *t* = 0 in neurons expressing TRPV4^WT^ and pre-treated for 4 hours with 10 μM KN-93 or KN-92, *n* = 44 (KN-92) and 28 neurons (KN-93) from 12 wells per genotype from three separate preparations. Two-way ANOVA, Geisser-Greenhouse correction (*p* < 0.0001), Tukey’s post hoc test. For all panels: *p < 0.05,**p < 0.01, ***p < 0.001.

To evaluate if TRPV4 activity alters intracellular Ca^2+^ dynamics in neurons in vivo, we co-expressed the genetically encoded calcium indicator GCaMP6s^23^ with TRPV4(mod) variants in C4da neurons. We analyzed Ca^2+^ dynamics by measuring changes in GCaMP6s fluorescence intensity in C4da neuronal somata of dissected third instar larvae after administration of vehicle or the TRPV4-selective agonist GSK1016790A (GSK101)^32,33^. C4da neurons expressing TRPV4^R269C^(mod) demonstrate a faster Ca^2+^ response upon GSK101 application than C4da neurons expressing TRPV4^WT^(mod), with a time to half maximum intensity (*t*_1/2__max_) of 22 ± 2.4 s (best fit parameter ± SEM) for TRPV4^R269C^ compared with a *t*_1/2__max_ of 41 ± 3.0 s for TRPV4^WT^ (*p* < 0.0001) (Fig. 5a, b). There is also a trend toward a higher maximum response in TRPV4^R269C^-expressing larvae (Fig. 5a, b). Control C4da neurons expressing no TRPV4 or TRPV4^R269C+M680K^ show no change in Ca^2+^ levels in response to GSK101, and no neurons responded to DMSO treatment alone (Fig. 5a, b, Supplementary Fig. 7a). We also monitored spontaneous calcium transients in the larval ventral nerve cord using myristoylated GCaMP5 in C4da neurons expressing TRPV4 variants. Notably, C4da neurons expressing TRPV4^R269C^(mod) have a marked increase in spontaneous calcium transients in axonal projections compared with control neurons and neurons expressing TRPV4^WT^ (Supplementary Fig. 7b and Supplementary Videos 1–3). This increase is abolished by inclusion of the M680K mutation (Supplementary Fig. 7b and Supplemental Video 4), suggesting that the increase in calcium transient frequency is TRPV4 channel pore-dependent.

To determine whether similar effects are observed in mammalian neurons, we cultured primary mouse trigeminal neurons and transduced them with equivalent titers of lentiviral vectors encoding TRPV4^WT^-EGFP or TRPV4^R269C^-EGFP (Supplementary Fig. 8a, b). Both TRPV4^WT^-EGFP and TRPV4^R269C^-EGFP express at equivalent levels and localize to the cell cortex in the somata and neurites (Supplementary Fig. 8c, d). We determined somal Ca^2+^ signals by measuring the Fura-2AM 340/380 ratio. As predicted based on calcium imaging of nonneuronal cells^5^, there is a small but statistically significant increase in baseline calcium levels in neurons expressing TRPV4^R269C^ compared with TRPV4^WT^ (Supplementary Fig. 8e). Like fly neurons, mouse trigeminal neurons transduced with TRPV4^R269C^-EGFP respond more rapidly and more robustly to GSK101 with a *t*_1/2__max_ of 16 ± 3.8 s (best fit parameter ± SEM) as compared with TRPV4^WT^ (28 ± 8.5 s) (Fig. 5c, d). The maximum response of TRPV4^R269C^ (1.2 ± 0.016) is greater than that of TRPV4^WT^ (0.91 ± 0.014) (*p* < 0.0001). Collectively, these data indicate that the R269C mutation sensitizes the TRPV4 channel in *Drosophila* neurons in vivo and primary mammalian neurons in vitro, consistent with the gain-of-ion channel function phenotypes observed in flies.

To determine whether CaMKII is required for TRPV4-induced Ca^2+^ influx, we treated primary trigeminal neurons expressing TRPV4^WT^-EGFP with the calmodulin-targeting CaMKII inhibitor KN-93 or its inactive analog KN-92^34,35^. Remarkably, KN-93 significantly attenuates Ca^2+^ responses to GSK101 in neurons expressing TRPV4^WT^-EGFP, whereas those treated with KN-92 respond normally to agonist stimulation (Fig. 5e). We observe similar inhibitory effects when treating with autocamtide-2-related inhibitory peptide (AIP), a small peptide CaMKII inhibitor^36^ (Supplementary Fig. 9a). The effect of CaMKII inhibition is not owing to altered TRPV4 surface expression, as CaMKII inhibition does not alter the cortical localization of TRPV4-EGFP (Supplementary Fig. 9b, e). Furthermore, CaMKII inhibition with KN-93 also prevents increases of intracellular Ca^2+^ levels mediated by endogenous TRPV4 in an immortalized rat dorsal root ganglion cell line^37^ (Supplementary Fig. 10a, c). These data indicate that CaMKII is required for TRPV4-mediated increases in intraneuronal Ca^2+^ levels.

### TRPV4^R269C^ disrupts mitochondrial axon transport

**Fig. 6:**
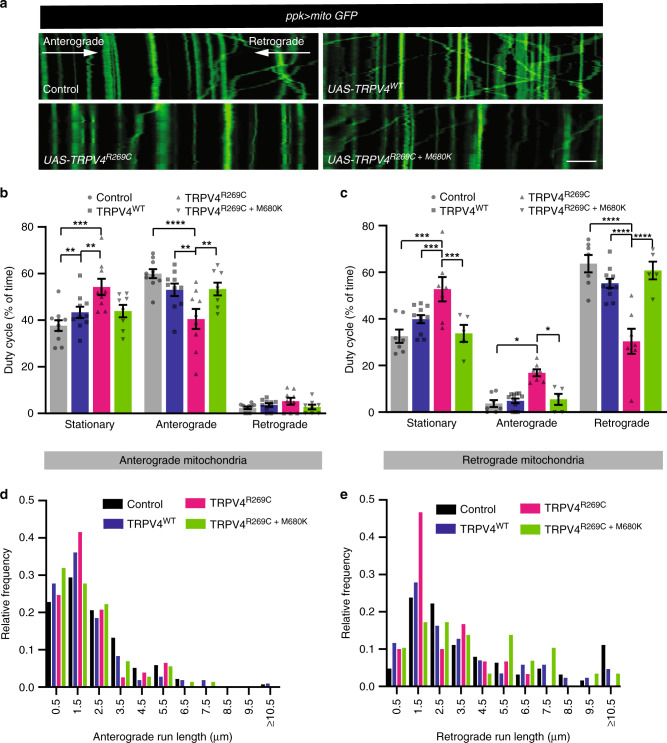
Mitochondrial axonal transport is disrupted by TRPV4^R269C^ prior to axonal degeneration. **a** Kymographs of mito-GFP transport in the proximal axon of C4da neurons expressing the indicated TRPV4(mod) variants. Scale bar, 10 μm. Quantification of **b** anterograde duty cycle and **c** retrograde duty cycle. For control, TRPV4^WT^, TRPV4^R269C^, TRPV4^R269C+M680K^ *n* = 10, 10, 9, 8 and 7, 11, 7, 5 larvae for anterograde and retrograde, respectively. Mean ± SEM. Two-way ANOVA (*p* < 0.0001), Tukey’s post hoc test. Frequency distributions of **d** anterograde and **e** retrograde mitochondrial run lengths. For control, TRPV4^WT^, TRPV4^R269C^, TRPV4^R269C+M680K^: *n* = 12, 11^,^ 14, and 10 larvae; 138, 108, 77, and 72 anterograde and 63, 86, 30, and 29 retrograde runs, respectively. For all panels: *p < 0.05, **p < 0.01, ***p < 0.001, ****p < 0.0001.

Axonal transport of mitochondria is regulated by intracellular Ca^2+^ ^9,10,38^. To assess mitochondrial axon transport dynamics, we imaged the proximal axon of C4da neurons co-expressing TRPV4(mod) variants and the mitochondrial reporter mito-GFP. We used the TRPV4^R269C^(mod) line as it does not exhibit observable morphological phenotypes in C4da neuron axons or their central projections (Supplementary Fig. 3d, e). TRPV4^R269C^(mod) expression significantly inhibits mitochondrial axon transport compared with controls expressing no TRPV4, whereas TRPV4^WT^(mod) and TRPV4^R269C+M680K^(mod) has minimal impact. Specifically, both anterograde and retrograde axonal mitochondria are more often stationary (Fig. 6a, c), and retrograde more than anterograde mitochondria have shorter run lengths (Fig. 6d, e).

**Fig. 7:**
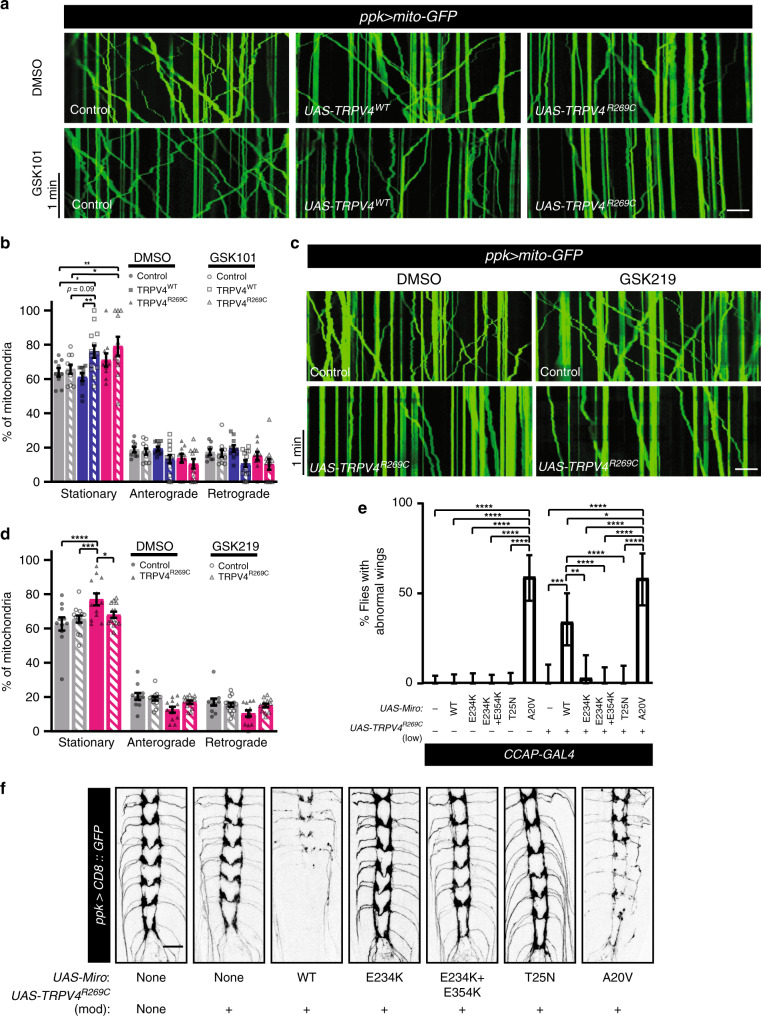
Pharmacologic manipulation of TRPV4 modulates mitochondrial transport and the mitochondrial transport protein Miro enhances TRPV4^R269C^-mediated toxicity. **a** Kymographs of mito-GFP transport in C4da neuron proximal axons in larvae expressing no TRPV4, TRPV4, or TRPV4^R269C^ treated with 40 nM GSK101 or DMSO. Scale bar, 10 μm. **b** Fraction of stationary, anterograde-, and retrograde-moving mitochondria from **a**. Mean ±SEM. For control, TRPV4^WT^, and TRPV4^R269C^: DMSO *n* = 9, 10, 10 larvae; GSK101 *n* = 10, 14, 11 larvae. Two-way ANOVA (*p* < 0.0001), Tukey’s post hoc test. **c** Kymographs of mito-GFP transport in C4da neuron axons in larvae expressing no TRPV4 or TRPV4^R269C^ raised on food containing DMSO or 100 µM GSK219 and treated with 10 µM GSK219 during imaging. Scale bar, 10 μm. **d** Percentage of stationary, anterograde-, and retrograde-moving mitochondria from **c**. Mean ± SEM. For control and TRPV4^R269C^: DMSO *n* = 11 and 12 larvae; GSK219 *n* = 14 and 13. Two-way ANOVA (*p* < 0.0001), Tukey’s post hoc test. **e** Percentage ± 95% CI of flies with unexpanded wings when expressing Miro variants ± TRPV4^R269C^(low) in N_CCAP_. From left to right *n* = 86, 72, 64, 77, 62, 54, 33, 38, 32, 39, 35, and 41 flies. *Χ*^2^ test (*p* < 0.0001) followed by pairwise two-sided Fisher’s exact test. **f** Representative confocal images of C4da neuron projections in larvae expressing TRPV4^R269C^(mod) (as in Supplementary Fig. 2d and e) ± overexpressed Miro variants. Similar results observed in all imaged larvae within each genotype (*n* = 7, 8, 8, 8, 7, 8, and 8 larvae from left to right) Scale bar, 25 μm. For all panels: *p < 0.05, **p < 0.01, ***p < 0.001, ****p < 0.0001.

To test whether pharmacologic activation of TRPV4^WT^ is sufficient to disrupt mitochondrial transport dynamics, TRPV4^WT^(mod) larvae were treated with GSK101. This results in an increased proportion of stationary mitochondria and reduced run length similar to that observed in animals expressing TRPV4^R269C^(mod) alone (Fig. 7a, b, Supplementary Fig. 11a, b and d, e). Interestingly, larvae expressing TRPV4^R269C^ exhibit minimal further disruption of mitochondrial transport in response to GSK101 (Supplementary Fig. 11c, f), possibly owing to saturation of the molecular machinery responsible for inhibiting transport. We also tested whether treating TRPV4^R269C^(mod)-expressing larvae with the TRPV4-selective antagonist GSK219 can ameliorate mitochondrial transport defects. GSK219 normalizes the proportion of motile mitochondria (Fig. 7c, d) and causes a shift to longer retrograde run lengths in TRPV4^R269C^-expressing larvae, but not control larvae (Supplementary Fig. 11g–j). These data indicate that TRPV4 activation, induced either by the R269C mutation or by a pharmacological agonist, disrupts mitochondrial axon transport.

### Miro modifies TRPV4^R269C^-mediated toxicity

Notably, the TRPV4-mediated axonal mitochondrial transport phenotypes (Figs. 6, 7a–b) are similar to those seen in *Drosophila* larvae with homozygous loss-of-function mutations of mitochondrial Rho (Miro)^39,40^. Miro is a Ca^2+^-binding GTPase that localizes to the outer membrane of mitochondria and regulates mitochondrial coupling to microtubule motors^38,41,42^. Miro binds Ca^2+^ via two EF hand domains, and this Ca^2+^ binding promotes the dissociation of mitochondria from the microtubule motor or microtubule. The amino-terminal GTPase domain of Miro is required for appropriate transport of mitochondria along axons^39,40,43^, though the specific function of this domain remains unknown. To test whether Miro and TRPV4 function in a common pathway, we tested for genetic interactions between TRPV4^R269C^ and Miro variants in N_CCAP_ and C4da neurons. While TRPV4^R269C^(low) or wild type Miro do not cause a wing phenotype when expressed individually, co-expression of both Miro and TRPV4^R269C^(low) cause an ~40% penetrant wing phenotype (Fig. 7e). Interestingly, expression of a GTP-bound Miro mutant (Miro^A20V^) alone is sufficient to cause impaired wing expansion, and this phenotype is not enhanced by co-expression of TRPV4^R269C^ (Fig. 7e). Similarly, co-expression of Miro^WT^ or Miro^A20V^ together with TRPV4^R269C^(mod) causes axonal degeneration in C4da neurons (Fig. 7f). Overexpression of Miro^WT^ alone only mildly alters C4da neuron central projections, whereas Miro^A20V^ alone is sufficient to cause axonal degeneration (Supplementary Fig. 12a). In contrast, expression of a Miro variant locked in the GDP-bound confirmation (Miro^T25N^) suppresses the TRPV4^R269C^(mod)-mediated wing phenotype and does not cause N_CCAP_ toxicity when co-expressed with TRPV4^R269C^(low) (Supplementary Fig. 12b). In addition, expression of Miro^T25N^ suppresses axonal degeneration in C4da neurons induced by TRPV4^R269C^(high) and does not cause toxicity in C4da neurons when co-expressed with TRPV4^R269C^(mod) (Supplementary Fig. 12c–d). These data suggest that GTP binding of the amino-terminal GTPase domain of Miro is necessary and sufficient to promote neurotoxicity downstream of TRPV4 activation.

Notably, these genetic interactions are not observed in N_CCAP_ with Miro variants unable to bind Ca^2+^ (Miro^E234K^ or Miro^E234K+E354K^) (Fig. 7e). Moreover, Miro variants that cannot bind Ca^2+^ do not induce C4da axonal degeneration when co-expressed with TRPV4^R269C^(mod) (Fig. 7f). Thus, Miro-mediated enhancement of TRPV4^R269C^-dependent axonal degeneration is dependent on the ability of Miro to bind Ca^2+^, suggesting that TRPV4^R269C^ causes axonal degeneration by promoting the binding of Ca^2+^ to Miro.

## Discussion

Inherited motor and sensory peripheral neuropathy, also known as CMT, is the most common form of genetically determined neuromuscular disease^21^. Despite the identification of over 100 causative genes, treatment remains elusive. TRPV4 is one of the rare ion channels that can directly cause peripheral nerve degeneration^1–3^, yet the mechanisms leading to neuronal dysfunction are unknown. In this study, we demonstrate in *Drosophila* and primary mammalian neurons that a neuropathy-causing mutant, TRPV4^R269C^, causes neurodegeneration and increases intraneuronal Ca^2+^ via a mechanism that requires a functional ion channel pore (Supplementary Fig. 13). In a genetic screen, we identified CaMKII, a Ca^2+^-dependent kinase, as a potent modifier of TRPV4^R269C^-mediated neurotoxicity, unveiling an important role for CaMKII in neuronal TRPV4-associated Ca^2+^ signaling. Futhermore, we show that impaired mitochondrial trafficking and the Ca^2+^-binding protein Miro are important determinants of neurotoxicity downstream of TRPV4 activity.

Dominant missense mutations in *TRPV4* cause a spectrum of in vivo neuropathies that can present congenitally or late in adulthood^1–3^. In this study, we observed cell-autonomous neuronal dysfunction and/or degeneration in multiple *Drosophila* neuronal subtypes as a consequence of expressing a neuropathogenic mutant form of TRPV4. These phenotypes manifest as early as late larval developmental stages, when abnormalities in neuronal development or maintenance may be relevant, but we also demonstrate that mutant TRPV4 expression can cause adult onset, progressive neuronal dysfunction. Phenotype severity is dependent on TRPV4 expression levels, suggesting that variations in the timing and/or level of TRPV4 expression may be relevant determinants of the marked disease heterogeneity observed in patients.

Neuronal dysfunction and degeneration caused by neuropathogenic TRPV4 mutants in vivo were dependent on a functional channel pore, consistent with a gain-of-function mechanism of toxicity. These observations are consistent with prior data in cultured cell lines^1,2,5,6^ and the lack of neurodegenerative phenotypes in *Trpv4*-null mice^44,45^. Furthermore, small molecule inhibition of TRPV4 ameliorates TRPV4-mediated phentoypes in *Drosophila*, suggesting that TRPV4 antagonism is a promising therapeutic strategy for the treatment of patients with TRPV4-associated neuropathies. TRPV4 inhibition has also been shown to be protective in rodent models of chemotherapy-induced peripheral neuropathy^46,47^, suggesting that TRPV4 antagonists may have utility in peripheral neuropathies of diverse etiologies. This is particularly exciting as small molecule TRPV4 antagonists have already proven to be safe in clinical trials for pulmonary edema in heart failure^7^.

Our study highlights that increased intracellular Ca^2+^ is a fundamental consequence of TRPV4^R269C^ activation in *Drosophila* neurons in vivo and in mammalian neurons in vitro. Tightly regulated Ca^2+^ homeostasis is critical to myriad aspects of normal neuronal physiology and may be disrupted during neurodegeneration. To identify specific pathways acting downstream of TRPV4^R269C^, we conducted, to our knowledge, the largest genetic modifier screen in a *Drosophila* model of CMT and identified CaMKII as the most potent modifier. We further demonstrated that inhibition of CaMKII ameliorates multiple mutant TRPV4-mediated neuronal phenotypes in flies and also dramatically suppresses TRPV4-mediated intracellular Ca^2+^ responses in mammalian neurons.

CaMKII is a critical Ca^2+^-sensitive kinase that transduces changes in intracellular Ca^2+^ to regulate diverse neuronal processes, including neurite morphogenesis, neuronal excitability, synaptic plasticity, and ion channel activity^28,48^. We envision two possible mechanisms by which CaMKII influences TRPV4-mediated increases in intracellular Ca^2+^. First, CaMKII could directly modulate TRPV4 channel gating. Indeed, the gating properties of ion channels such as AMPA receptors can be strongly modulated by CaMKII-dependent phosphorylation^49^. CaMKII is a putative binding partner of TRPV4^50,51^, consistent with the possibility that TRPV4 is directly regulated by CaMKII, but, to our knowledge, no CaMKII-dependent phosphorylation sites in TRPV4 have been identified.

A second potential mechanism is that activation of CaMKII by TRPV4-mediated Ca^2+^ influx could regulate subsequent TRPV4-independent Ca^2+^ release, either by opening of Ca^2+^-permeable channels within the plasma membrane or by activating release of intracellular Ca^2+^ stores. Several studies suggest that TRPV4 stimulation orchestrates activation of Ca^2+^-sensitive plasma membrane channels that amplify and propagate Ca^2+^ signaling events, and some of these responses can be modulated by inhibition of CaMKII^52–57^. In addition, TRPV4-mediated intracellular Ca^2+^ responses can be blunted by depleting endoplasmic reticulum (ER) Ca^2+^ stores or by inhibiting ER Ca^2+^ release channels, suggesting amplification of signaling by Ca^2+^-mediated ER Ca^2+^ release^58–60^. CaMKII can phosphorylate and regulate mammalian IP_3_ receptors and ryanodine receptors^61^ potentially linking activation of CaMKII with downstream ER Ca^2+^ release. Notably, our secondary genetic screen found partial suppression of the wing expansion phenotype with RNAi targeting the *Drosophila* ryanodine receptor (RyR) (Supplementary Data 2). Regardless of the mechanism by which CaMKII influences TRPV4-mediated intracellular Ca^2+^ elevations, our results highlight CaMKII as a potent modifier of TRPV4-mediated Ca^2+^ signaling and toxicity and add to the growing body of evidence underscoring key roles for CaMKII in neuronal Ca^2+^ homeostasis and degeneration^62–64^.

Transport of mitochondria within axons is a Ca^2+^-regulated process that is crucial to neuronal homeostasis. Disrupted mitochondrial transport is a common feature across many neurodegenerative diseases, although whether such disruption is an early pathogenic process or a reflection of non-specific neuronal dysfunction is unclear^11,12^. In this study, we observed mitochondrial transport defects downstream of neuropathogenic TRPV4 activity prior to the onset of observable degeneration, suggesting that mitochondrial transport impairments are an early pathological event. In addition, mitochondrial transport is impaired by acute activation of TRPV4^WT^, demonstrating a close temporal association between TRPV4 activation and impaired mitochondrial transport. As increased intracellular Ca^2+^ is sufficient to inhibit the axonal transport of mitochondria^38^, our data are consistent with a model in which TRPV4-mediated Ca^2+^ influx serves to regulate the function of axonal mitochondrial transport machinery. In animal models of chemotherapy-induced neuropathy, in which mitochondrial trafficking defects are well-established, inhibition of TRPV4 activity is partially protective^46,47^. Our results suggest that blocking TRPV4-mediated disruption of mitochondrial transport may be the mechanism for this protective effect.

Our data highlight the importance of the mitochondrial GTPase Miro, which regulates mitochondrial transport in fly and mammalian neurons via mechanisms that rely on the Ca^2+^-binding EF hand domains and amino-terminal GTPase domain, and suggest that Miro provides the link between TRPV4-mediated Ca^2+^ elevations and disrupted mitochondrial transport^38–40,43^. We observe a marked enhancement of TRPV4^R269C^-mediated neurotoxicity by overexpression of wild type Miro, but not by Miro that is unable to bind Ca^2+^, and the GTP-bound form of Miro recapitulates both the wing-opening failure and sensory neuron degenerative phenotypes, independent of TRPV4 expression. Together, these results suggest that dysregulation of Miro is a fundamental downstream effect of TRPV4 activation, as Miro disruption is both necessary and sufficient for the neuronal phenotypes we observe in our TRPV4 fly model. These results are in agreement with prior work highlighting the critical importance of Miro for neuronal health and survival. *miro*-null flies exhibit severe mitochondrial axon transport defects and early lethality^39^, whereas *Miro1*-null mice have degenerative motor neuron disease^65^.

Our results linking mitochondrial trafficking impairment and neuronal degeneration closely parallel findings in animal models harboring *MFN2* mutations that cause CMT2A. Similar to Miro, MFN2 has myriad roles in mitochondrial biology, and pathogenic *MFN2* mutations result in defective mitochondrial fusion and transport^14,15,66^. MFN2 agonists restore mitochondrial trafficking defects in pre-clinical models of CMT2A^67^, thus establishing a precedent for correcting defects similar to those reported in this study through small molecule manipulation of a mitochondrial GTPase. Moreover, a recent study suggests that CMT2 mutations in both MFN2 and TRPV4 prolong inter-mitochondrial contacts^68^. Further investigation of functional interactions between TRPV4 and mitochondrial biology will help elucidate the precise contributions of these potential pathological mechanisms and could refine therapeutic strategies for a range of neuropathies.

In summary, our data support a model in which neuropathogenic TRPV4 mutants cause cell-autonomous neurotoxicity through a gain-of-TRPV4 ion channel function. This results in a CaMKII-dependent increase in neuronal Ca^2+^ that disrupts mitochondrial transport and causes axonal degeneration (Supplementary Fig. 13). These phenotypes are prevented by TRPV4 antagonists, which hold promise as a therapeutic strategy for the treatment of patients with TRPV4-associated neuropathies.

## Methods

### *Drosophila* stocks and husbandry

Flies were raised on a standard cornmeal-molasses food. All experiments were performed at 25 °C with a 12 hour/12 hour day/night cycle, unless otherwise noted. The following stocks were obtained from the Bloomington Stock Center: UAS-CD8::GFP, UAS-mito-GFP, ppk-GAL4, CCAP-GAL4, UAS-GCaMP6s, UAS-miro, UAS-CaMKII RNAi, UAS-CaMKII, UAS-CaMKII^T287D^, and UAS-CaMKII^T287A^. UAS-CaMKII RNAi lines (38940, 47280, and 100265) were also obtained from the Vienna *Drosophila* Research Center. UAS-miro^T25N^, UAS-miro^A20V^, UAS-miro^E234K^, and UAS-miro^E234K, E354K^ were kind gifts from Konrad Zinsmaier.

### Generation of human TRPV4-expressing *Drosophila* stocks

The human TRPV4 open reading frame (ORF) was PCR amplified from full-length human TRPV4 cDNA in pcDNA3.1 (WT, R269C, R269C + M680K, R232C)^1^ using primers (GGGGACAAGTTTGTACAAAAAAGCAGGCTTCACCATGGCGGATTCCAGCGAAGGC and GGGGACCACTTTGTACAAGAAAGCTGGGTCCTAGAGCGGGGCGTCATCAGTCCTCCA) and recombined into pDONR 221 using BP Clonase (Thermo Fisher Scientific). The TRPV4 ORF flanked by attL sites in the resultant entry vectors was fully sequenced and then recombined with either pBID^29^ or pTW vectors (*Drosophila* Gateway Vector Collection, Carnegie Institution for Science) using LR Clonase (Thermo Fisher). TRPV4(low) lines were generated by microinjection of pBID-UASC-hTRPV4 into M{vas-int.Dm}ZH-2A, y[1]; P{y[+]=CaryP}attP2 embryos by BestGene for PhiC31 site-specific integration at the *attP2* site. TRPV4(mod) and TRPV4(high) transgenic lines were microinjected into *w1118* embryos for random *P*-element insertion. Transgenic lines were identified and genetic elements mapped using conventional methods. TRPV4 mRNA and protein expression levels in each transgenic line were determined by reverse transcription quantitative PCR (RT-qPCR) and western blot analyses, respectively (see Methods below).

### Wing expansion assay

Flies carrying the TRPV4 transgene or controls were crossed to *w*^*1118*^;*CCAP-GAL4*/TM6B. Flies were transferred to new vials every 3–4 days. After two transfers, the P_0_ flies were discarded. Progeny were scored at two times from each vial: 2 and 4 days post eclosion of the first progeny from the cross to eclose. We selected flies carrying the *CCAP-GAL4* driver and transgene of interest and scored their wing phenotype by eye using a stereomicroscope. Flies wings were scored as fully expanded, partially expanded, or fully unexpanded. For analysis, partially expanded flies were counted as 0.5 normal and 0.5 unexpanded. Flies that had phenotypic traits of virgins at the time of initial collection were set aside and scored at least 4 hours later.

### TRPV4^R269C^ genetic modifier screen

Flies of the genotype *w*^*1118*^;*CCAP-GAL4*, *UAS-TRPV4*^*R269C*^*(mod)*/*TM6B, GAL80* were crossed to fly lines containing transposable elements designed to either knockdown or overexpress endogenous *Drosophila* genes downstream of the GAL4-binding UAS site (Supplementary Data 1 and 2). Putative modifier genes were selected based on their potential to be druggable^27^, CMT genes, and other genes implicated in TRPV4 signaling. We selected for flies carrying *CCAP*-GAL4, *UAS*-*TRPV4*^*R269C*^, and the screen element and scored their wing phenotypes as described above. For experiments studying genetic interactions with TRPV4^R269C^(low), flies of the genotype *w*^*1118*^*; CCAP-GAL4/ CyO; UAS-TRPV4*^*r269C*^*(low)/TM6B* were used.

### Climbing assays

We crossed our transgenic TRPV4(high) lines to flies carrying the *elav-GeneSwitch* driver. P_0_ flies were transferred to new vials every 3–4 days and were discarded after the second transfer. Progeny were allowed to develop on standard food. Upon eclosion, we selected flies carrying TRPV4 transgenes and the *elav-GeneSwitch* driver. Zero- to 3-day old flies were then transferred onto food containing 200 μM RU486 (Sigma) at a density of 10 flies per vial. Flies were transferred onto fresh RU486 food every 2 days. Climbing behavior was scored by transferring flies into a climbing assay chamber which was composed of two vials without food stacked on top of one another such that the two openings faced one another. Lines were drawn on the vials 8 cm from either end. Once flies were in the vial, the vial was tapped swiftly four times on the laboratory bench so as to knock all flies to the bottom. Flies were then allowed to climb for 10 seconds at which point we counted the number of flies above and below the 8 cm line. Flies were allowed to rest for 1 minute and the process was repeated for a total of 10 trials per vial of 10 flies.

### RT-qPCR and western blot analyses

Analyses of TRPV4 mRNA and protein levels were performed utilizing adult flies expressing TRPV4 variants selectively in neurons (*C155*-*GAL4* pan-neuronal driver). Heads were collected from adult flies at 1–3 days post eclosion and stored at −80 °C. For quantification of TRPV4 transcript levels, heads were homogenized in Trizol (ThermoFisher) using plastic pestles and RNA isolated using the RNeasy mini kit (Qiagen) including on-column DNase digestion. Following cDNA conversion utilizing the High Capacity cDNA Reverse Transcription kit (ThermoFisher), RT-qPCR was performed with the HT7900 Real-Time PCR system (ThermoFisher) using Taqman Universal PCR master mix and the following Taqman assays: human TRPV4 exons 3–4 (Hs01099348_m1), human TRPV4 exons 5–6 (Hs00540967_m1), human TRPV4 exons 7–8 (Hs00222101_m1), *Drosophila* RpII140 (Dm02134593_g1; all from ThermoFisher). For western blot analyses, samples were lysed in RIPA buffer (Sigma) supplemented with protease inhibitors (Cell Signaling Technology) and sonicated. Protein lysates were resolved on 4–15% TGX gels (Bio-Rad) and transferred to PVDF membranes (ThermoFisher). Primary antibodies used were polyclonal rabbit anti-TRPV4 (1:500; Abcam; ab39260), monoclonal rabbit anti-β-actin (1:1000; Cell Signaling Technology; #4970), and monoclonal mouse anti-GAPDH (1:5000; ThermoFisher; #AM4300), followed by HRP-conjugated mouse anti-rabbit (1:150,000; Jackson ImmunoResearch; #211-032-171) or goat anti-mouse (1:150,000; Jackson ImmunoResearch; #211-032-171) secondary antibodies. Membranes were developed using SuperSignal West Femto Maximum Sensitivity Substrate (ThermoFisher) and imaged using an ImageQuant LAS 4000 system (GE Healthcare).

### Larval dissections and immunostaining

Larvae were filleted in HL3 solution to expose the CNS and body wall, prior to fixation in 4% paraformaldehyde (PFA) in PBS for 20 minutes at room temperature with gentle shaking. Preparations were then washed three times over 10 minutes in PBS and blocked for 1 hour at room temperature in 5% normal goat serum in PBS with 0.1% Triton X-100 (PBST). Fillets were then incubated with primary antibody for 2 hours at room temperature or overnight at 4 °C in 5% normal goat serum in PBST. Primary antibodies included mouse anti-GFP IgG2a (1:1000, ThermoFisher, A-11120) and rabbit anti-GFP (1:1000, ThermoFisher, A-11122). They were then washed three times over the course of 1 hour with PBST and were then incubated for 1 hour with secondary antibody in 5% normal goat serum in PBST. Secondary antibodies included DyLight 488-conjugated goat anti-mouse IgG2a (1:1000, Jackson ImmunoResearch, 115-285-206) and Alexa Fluor 488-conjugated goat anti-rabbit IgG (1:1000, ThermoFisher, A-11034). Fillets were then mounted onto glass slides in Fluoromount-G (Southern Biotech) or Vectashield (Vector Labs) mounting media.

### Ca^2+^ imaging in *Drosophila* dendritic arborization neurons

120 h AEL third instar larvae expressing GCaMP6s were pinned ventral side up on silicone elastomer plates and dissected in external saline solution composed of: NaCl 120 mM, KCl 3 mM, MgCl_2_ 4 mM, CaCl_2_ 1.5 mM, NaHCO_3_ 10 mM, trehalose 10 mM, glucose 10 mM, TES 5 mM, sucrose 10 mM, and HEPES 10 mM. The osmolality was 305 mOsm kg^−1^, and the pH was 7.25. Internal debris was removed with forceps and the body wall pinned flat. Time-lapse imaging was performed under a water-immersion objective lens (W Plan-Apochromat ×20/1.0 DIC CG = 0.17 M27 75 mm) using a Zeiss LSM-700 confocal microscope. Frame rate was 1.27 Hz. Regions of interest (ROIs) were drawn around entire somata for trace examples. 5 µm diameter circle ROIs were placed on brightest part of each soma to calculate responses to 40 nM GSK101 or DMSO, which were added directly to the recording media.

Imaging of sponataneous calcium transients with GCaMP5-myr was performed on a Zeiss Axio-Observer widefield micrscope using a ×63 oil immersion lense. Larvae were dissected ventral side down in HL3 with 2.0 mM Ca^2+^ on a sylgard block. After removal of the internal tissues, the sylgard block to which the fillet was pinned was inverted and placed down on a large, circular glass coverslip such that the fillet rested against the coverslip while immersed in HL3. Images were acquired at a rate of four frames per second for one minute with excitation from a 475 nm LED light source. The acquired image series were then blinded, and an experimenter who was not involved in image acquisition counted the number of calcium transients, defined as clear, observable increases in GCaMP5-myr fluorescence intensity throughout each movie.

### Mouse husbandry

All mice used in this study were housed and handled according to protocols approved by the Animal Care and Use Committee of Johns Hopkins University School of Medicine.

### Mouse trigeminal neuron isolation and culture

Six to 10 week-old C57BL/6 J mice were deeply anesthetized with isoflurane and killed by cervical dislocation. The bilateral trigeminal ganglia were then harvested and dissociated via consecutive incubations in Hibernate (Brain Bits) with papain (37 °C 20 min, gentle agitation at 10 min) followed by collagenase/dispase (37 °C 20 min, gentle agitation at 10 min)^69^. After enzyme incubations, harvested ganglia were gently titruated 2–3 time with a P1000 pipette tip followed by an additional 2–3 passes through a P200 pipette tip. Titurated ganglia were carefully overlayed on a Percoll gradient and centrifuged at 1500 × *g* for 20 min. The top layer was carefully removed and centrifuged for 10 min at 1000 × *g.* Trigeminal neurons were plated on poly-l-ornithine and collagen/laminin coated eight-well Borosilicate coverglass imaging chambers (ThermoFisher). Neurons were transduced with virus carrying GFP, TRPV4^WT^-EGFP, or TRPV4^R269C^-EGFP 24 hours after plating. Experiments were performed 72 hours after viral transduction.

### Lentivirus production

TRPV4^WT^ and TRPV4^R269C^ with a C-terminal EGFP tag in pcDNA3.1 were subcloned into the lentivirus transfer vector FUGW (gift from David Baltimore, Addgene plasmid #14883) (PMID 11786607). In brief, TRPV4-EGFP was PCR amplified (Forward primer: CTTGGGCTGCAGGTCGACTCTAGAGATGGCGGATTCCAGCGAAG, Reverse primer: TTGATTATCGATAAGCTTGATATCGTTACTTGTACAGCTCGTCCATG) and subcloned into the EcoRI and BamHI restriction sites of the FUGW plasmid using the Gibson Assembly Cloning Kit (New England Biolabs). Lentivirus was produced by transfecting HEK293T cells with second generation packaging vectors pCMV-VSV-G and pCMV-dR8.91 (gifts from Jeffrey Rothstein) along with FUGW TRPV4-EGFP (WT or R269C). HEK293T cells were transfected using polyethylenimine (PEI) and virus-containing media was harvested at 48 and 72 hours post transfection and concentrated using Lenti-X Concentrator (Takara).

### Trigeminal neuron Ca^2+^ imaging

Virally transduced trigeminal neurons and 50B11 cells were loaded with the ratiometric Ca^2+^ indicator Fura-2AM (ThermoFisher) for 1 hour at 37 °C per the manufacturer’s instructions. Ca^2+^ imaging was performed on a Zeiss AxioObserver.Z1 inverted microscope equipped with a Lambda DG-4 (Sutter Instrument Company) wavelength switcher. Prior to Ca^2+^ imaging of trigeminal neurons, a single channel GFP image was acquired to measure TRPV4 expression within individual neurons. Cells were imaged at 340 nm and 380 nm excitation at three 10 second intervals to acquire a baseline fluorescence measurement prior to the application of the TRPV4-selective agonist GSK1016790A (MilliporeSigma), after which cells were imaged for ~4 minutes at a rate of one frame per 10 seconds. Baseline calcium was determined at *t* = −10 s, the timepoint immediately prior to addition of GSK1016790A. The CaMKII inhibitor KN-93 and its inactive analog KN-92 (Cayman Chemical) were used at 10 μM and applied for 4 hours prior to imaging. AIP (MilliporeSigma) was used at 10 μM and applied two hours prior to imaging.

### Trigeminal neuron fixation and immunostaining

Coverslips with transduced trigeminal neurons were washed three times quickly with PBS. Neurons were then fixed for 15 minutes at room temperature in 4% PFA. PFA was removed and coverslips were rinsed with PBS. Neurons were then permeabilized with PBS plus 0.1% TritonX-100 (PBST) for 10 minutes. Coverslips were then washed briefly with PBS and incubated with mouse anti-GFP IgG2a (1:1000, ThermoFisher, A-11122) and chicken anti-TUJ1 (1:1000) antibodies diluted in blocking buffer (4% normal goat serum in PBST) overnight with gentle shaking at 4 °C. Coverslips were then washed six times, 10 minutes each in PBST and were then incubated with DyLight 488-conjugated goat anti-mouse IgG2a (1:1000, Jackson ImmunoResearch, 115-285-206) and Alexa Fluor 555-conjugated goat anti-chicken (1:1000, ThermoFisher, A-21437) secondary antibodies in blocking buffer for 1 hour at room temperature with gentle shaking. Coverslips were then washed six times, 10 minutes each in PBST. Coverslips were than mounted with VectaShield+DAPI (Vector Labs) and sealed with nail polish.

### Confocal microscopy-fixed imaging

Slides were imaged on a Zeiss 800 LSM confocal laser scanning microscope with a ×20 air, ×40 oil immersion, or ×63 oil immersion objective. Image acquisition parameters were kept uniform across all samples in a given experiment.

### Confocal microscopy and live imaging of trigeminal neurons

Transduced trigeminal neurons were incubated for 2 hours with 10 μM KN-92 or KN-93. Neurons were then incubated for 20 minutes with ER-Tracker Red (0.5 μM, ThermoFisher, E34250) diluted in artificial cerebrospinal fluid (aCSF) with either KN-92 or KN-93. Wells were then washed quickly three times with pre-warmed aCSF, and cells were finally placed in pre-warmed aCSF with either 10 μm KN-92 or 10  μm KN-93. Neurons were imaged on a Zeiss 800 LSM confocal laser scanning microscope with a ×63 Plan-apochromat oil immersion lens.

### Live cell imaging of axonal transport in *Drosophila*

Mito-GFP was used to label mitochondria and was expressed in C4da neurons along with TRPV4 using the *ppk-GAL4* driver. Wandering third instar larvae were dissected in HL3 solution with 0.6 mM Ca^2+^ and 4 mM glutamate as described^70^. Larvae were then mounted in HL3 solution on glass slides with the coverslip secured via dental glue (Surgident, #50092189). For experiments involving drug treatments, drug was added to the dissection media and allowed to incubate at least 5 minutes prior to image acquisition. Imaging was performed on a Zeiss 800 LSM with a ×63 oil objective. Image series of C4da neuron axons were captured in a single focal plane at an acquisition rate of 1 frame/second for 2 minutes, in a 200 μm~500 μm window from the cell body in abnominal segements A6 and A7. Movement dynamics were calculated by tracking the position of individual mitochondria frame by frame in ImageJ as previously described^70^. Since one pixel represents 0.198 μm in our image acquisition, only net velocities >0.2 μm/s with more than three consecutive frames in one direction were considered as anterograde or retrograde. Moving mitochondria that could be tracked for >60 frames continuously were considered for measuring mitochondrial run length or duty cycle, and representative kymographs were generated from the acquired time-lapse images using Zeiss Zen Blue 2.3 software. For experiments involving GSK1016790A, larvae were bathed in recording media which contained GSK1016790A during mounting for imaging. For experiments involving GSK2193874, larvae were raised on food with 10 μM GSK2193874 due to the observed insensitivity of TRPV4^R269C^ to this compound in electrophysiologic recordings (Fig. 4c). Larvae were also bathed in recording media with 10 μM GSK2193874 during dissection and mounting for imaging.

### Electrophysiological recordings of N_CCAP_

Experiments were performed on 1–3-day old adult female flies. Perforated patch-clamp recordings with β-escin were performed as previously described with minor modifications^25^, in order to measure action potentials (APs) from CCAP neurons located in the brain. Brains were removed and dissected in a *Drosophila* physiological saline solution (101 mM NaCl, 3 mM KCl, 1 mM CaCl_2_, 4 mM MgCl_2_, 1.25 mM NaH_2_PO_4_, 20.7 mM NaHCO_3_, and 5 mM glucose; pH 7.2), which was pre-bubbled with 95% O_2_ and 5% CO_2_. To better visualize the recording site, the perineuronal sheath surrounding the brain was focally and carefully removed after treating with an enzymatic cocktail, collagenase (0.4 mg/ml) and dispase (0.8 mg/ml), at 22 °C for 1 minute and cleaning with a small stream of saline pressure-ejected from a large diameter pipette using a 1 mL syringe. In addition, prior to recording, cell surfaces were cleaned with saline pressure-ejected from a small diameter pipette, using a 1 ml syringe connected to the pipette holder. The recording chamber was placed on an X–Y stage platform (PP-3185-00; Scientifica, UK). The cell bodies of the targeted CCAP neurons were visualized with CD8:GFP fluorescence on a fixed-stage upright microscope (BX51WI; Olympus, Japan) and viewed with a ×40 water-immersion objective lens (LUMPlanFl, NA: 0.8, Olympus). Patch pipettes (8–12 MΩ) were fashioned from borosilicate glass capillaries without filaments (OD/ID: 1.2/0.68 mm, 627500, A-M systems, WA) using a Flaming-Brown puller (P1000; Sutter Instrument), and further polished with a MF200 microforge (WPI) prior to filling with the internal pipette solution (102 mM potassium gluconate, 0.085 mM CaCl_2_, 0.94 mM EGTA, 8.5 mM HEPES, 4 mM Mg-ATP, 0.5 mM Na-GTP, 17 mM NaCl; pH 7.2). Biocytin hydrazide (13 mM; ThermoFisher) was added to the pipette solution before the recording. Recordings were acquired with an Axopatch 200B amplifier (Molecular Devices) and sampled with a Digidata 1440 A interface (Molecular Devices). These devices were controlled via pCLAMP 10 software (Molecular Devices). The signals were sampled at 20 kHz and low-pass filtered at 2 kHz. Junction potentials were nullified prior to high-resistance (GΩ) seal formation. Cells showing evidence of “mechanical” breakthrough, as assessed by the abrupt generation of a large capacitance transient (as opposed to the more progressive, gradual one generated by chemical perforation) were excluded. One CCAP neuron per brain was recorded. During the recording, the bath solution was slowly but continuously exchanged with fresh saline by means of a gravity-driven system (approximate flow rate of 1–2 ml/min). The mean firing rate of spontaneous APs and RMP were determined by the mean value of voltage during zero-holding current (0 pA as *I*_hold_) gap-free recording. For quantifying intrinsic membrane excitability, APs were elicited in response to current injections with 300 ms stepping pulses at 20 pA increments up to 100 pA. Electrophysiological analysis of both spontaneous APs and evoked APs was performed using custom MATLAB-based software. APs were detected automatically by identification of local maxima and were then manually curated to remove excitatory post-synaptic potentials using minimum voltage threshold criteria. Frequency of detected APs was quantified as mean firing rate during current injection. The current threshold (minimum current to evoke spiking) and the slope of the *f–I* curve were determined by linear regression of the curve from the point of initial spiking. To analyze subthreshold membrane potential dynamics as Δramp, spontaneous APs were first removed from the raw traces by introducing median filter and smoothing using a 7–18 ms (depending on spike width) moving average. Δramp was then quantified as the difference between the most depolarized value and the most hyperpolarized value during the recording.

Internal Ca^2+^ chelation experiments were carried out by intracellular perfusion with the Ca^2+^ chelator, BAPTA. For experiments with intracellular perfusion of BAPTA, a conventional whole-cell patch technique was used. Patch pipettes filled with internal pipette solution containing 5 mM BAPTA were brought up to the cell bodies. After GΩ seal formation, the patched membrane was ruptured by applying negative pressure. Data acquisitions of membrane potential were initiated 5 min following the rupture of the membrane.

GSK2193874 was prepared as a 20 mM stock solution dissolved in DMSO, and this stock was mixed into *Drosophila* physiological saline solution at a final concentration of 100 nM, 500 nM, or 10 μM. To achieve substantial penetration of the drug into the whole-brain tissue preparation, data acquisitions of membrane potential were carried out after 60 min bath application of the drug as preincubation period. During the preincubation period, 95% O_2_ and 5% CO_2_ pre-bubbled *Drosophila* physiological saline solution containing the drug was continuously perfused by Peristaltic pump (MINIPULS 3, Gilson) with flow rate of 2–3 ml/min. Recovery was not tested because of the considerable amount of time required to wash out drugs, in particular hydrophobic compounds, such as GSK2193874.

After recording the physiological responses of N_CCAP_, biocytin hydrazide was iontophoresed into the cell with a constant hyperpolarizing current of 0.9–1.2 nA passed for at least 5 minutes. The brain was then fixed in 4% PFA in PBS overnight at 4 °C. After washing for 1 hour in several changes of PBST (0.3% Triton X-100 in PBS) at room temperature, the brain was incubated with a mouse anti-GFP antibody (Invitrogen, 1:200) for 16-40 hours on a shaker at 4 °C, followed by incubation with an Alexa Fluor 488-conjugated goat anti-mouse (Invitrogen, 1:1000) secondary antibody and Alexa Fluor 568-conjugated streptavidin (Invitrogen, 1:100) for 24–40 hours on a shaker at 4 °C. After a 1 hour wash, samples were cleared in 70% glycerol in PBS for 5 minutes at room temperature and then mounted in Vectashield (Vector Labs). Recorded CCAP neurons were imaged using a confocal imaging system (LSM-700; Carl Zeiss).

### Image analysis

Fluorescent images were analyzed using ImageJ (NIH) or Imaris (Bitplane, Zurich, Switzerland). Synaptic area measurements were acquired in ImageJ using the Analyze Particles tool. In brief, confocal stacks were converted to maximum intensity projections and background fluorescence subtracted using the Math function. The image was then thresholded and an ROI was drawn around the segment to be measured and CD8-GFP positive area was measured using the Analyze Particles tool. Dendritic branching was measured in ImageJ or Imaris. In ImageJ confocal stacks were converted to maximum intensity projections, background fluorescence was then subtracted and the image was thresholded and converted to a binary image. Branching was measured using the Sholl analysis tool. In Imaris, dendrites were semi-automatically traced using the Filaments tool. In brief, images were thresholded to identify the center and seed points, filaments were then manually evaluated and incorrected tracings were removed and missing tracings were added. Branching was assessed using the Sholl branching tool. In either software, the spacing of the Sholl radii was 10 μm.

### Statistical analysis

Statistical analyses were performed in Prism 7.0 (GraphPad). For normally distributed datasets comparing summative statistics (e.g., mean) an unpaired *t* test, or a one- or two-way analysis of variance (ANOVA) was used, as appropriate. For data sets of repeated measures (e.g., calcium imaging over time), a two-way ANOVA with a Geisser-Greenhouse correction was used. If the ANOVA detected a significant interaction (*α* = 0.05), Tukey’s post hoc test was performed to identify which groups were significantly different. The level of significance is indicated as *p < 0.05, **p < 0.01, ***p < 0.001, ****p < 0.0001. Sample sizes (“*n*”) and indicators of statistical significance are noted within the Figure legends. For all graphs of *Drosophila* wing expansion, data are presented as a percentage of whole. The error bars in those graphs depict the 95% confidence intervals that were calculated using the Wilson/Brown method. A *Χ*^2^ test across all experimental groups was performed to test for the presence of any interaction. Pairwise Fisher’s exact tests were then performed between relevant groups to assess for significant inter-group differences.

### Reporting summary

Further information on research design is available in the Nature Research Reporting Summary linked to this article.

## Supplementary information

Supplementary Information

Peer Review File

Supplementary Data 1

Supplementary Data 2

Supplementary Movie 1

Supplementary Movie 2

Supplementary Movie 3

Supplementary Movie 4

Reporting Summary

## Data Availability

All data supporting the findings of this study and unique biological materials used in this study are available from the corresponding authors upon reasonable request. The source data for Figs. 1a, e, f; 2b, d, g; 3c, e, f, g; 4b, c, e; 5b, d, e; 6b–e; 7b, d–f; and Supplementary Figs. 1a–c; 2c; 3c, e; 5a–i; 6a–c; 7a, b; 8a, b, d, e; 9a, c–e; 10a–c; 11a–j; 12b, d are provided in the Source Data file.

## Source data

Source Data

## Code availability

The computer code to produce the figures of this paper and the other findings of this study are available upon reasonable request. Custom code for MATLAB-based software used for electrophysiology analysis is available upon request from Dr. Masashi Tabuchi, Department of Neurosciences, Case Western Reserve University School of Medicine, 2210 Circle Dr, Cleveland, OH, 44106, USA, dorcusmasashi@gmail.com.

## References

[1] Landouré G (2010). Mutations in TRPV4 cause Charcot-Marie-Tooth disease type 2C. Nat. Genet..

[2] Deng H-X (2010). Scapuloperoneal spinal muscular atrophy and CMT2C are allelic disorders caused by alterations in TRPV4. Nat. Genet..

[3] Auer-Grumbach M (2010). Alterations in the ankyrin domain of TRPV4 cause congenital distal SMA, scapuloperoneal SMA and HMSN2C. Nat. Genet..

[4] Watanabe H (2002). Activation of TRPV4 channels (hVRL-2/mTRP12) by phorbol derivatives. J. Biol. Chem..

[5] Sullivan JM (2015). Novel mutations highlight the key role of the ankyrin repeat domain in *TRPV4* -mediated neuropathy. Neurol. Genet..

[6] Klein CJ (2011). TRPV4 mutations and cytotoxic hypercalcemia in axonal Charcot-Marie-Tooth neuropathies. Neurology.

[7] Goyal N (2019). Clinical pharmacokinetics, safety, and tolerability of a novel, first-in-class TRPV4 ion channel inhibitor, GSK2798745, in healthy and heart failure subjects. Am. J. Cardiovasc. Drugs.

[8] Spillane J, Kullmann DM, Hanna MG (2016). Genetic neurological channelopathies: molecular genetics and clinical phenotypes. J. Neurol. Neurosurg. Psychiatry.

[9] Hammerschlag R, Dravid AR, Chiu AY (1975). Mechanism of axonal transport: a proposed role for calcium ions. Science.

[10] Breuer AC, Atkinson MB (1988). Calcium dependent modulation of fast axonal transport. Cell Calcium.

[11] Millecamps S, Julien J-P (2013). Axonal transport deficits and neurodegenerative diseases. Nat. Rev. Neurosci..

[12] Prior R, Van Helleputte L, Benoy V, Van Den Den L (2017). Defective axonal transport: a common pathological mechanism in inherited and acquired peripheral neuropathies. Neurobiol. Dis..

[13] Züchner S (2004). Mutations in the mitochondrial GTPase mitofusin 2 cause Charcot-Marie-Tooth neuropathy type 2A. Nat. Genet..

[14] Baloh RH, Schmidt RE, Pestronk A, Milbrandt J (2007). Altered axonal mitochondrial transport in the pathogenesis of Charcot-Marie-Tooth disease from mitofusin 2 mutations. J. Neurosci..

[15] Misko A, Jiang S, Wegorzewska I, Milbrandt J, Baloh RH (2010). Mitofusin 2 is necessary for transport of axonal mitochondria and interacts with the miro/milton complex. J. Neurosci..

[16] Wong C-O (2014). A TRPV channel in drosophila motor neurons regulates presynaptic resting Ca2+ levels, synapse growth, and synaptic transmission. Neuron.

[17] Peabody NC (2008). Bursicon functions within the drosophila CNS to modulate wing expansion behavior, hormone secretion, and cell death. J. Neurosci..

[18] Luan H (2006). Functional dissection of a neuronal network required for cuticle tanning and wing expansion in Drosophila. J. Neurosci..

[19] Deng Z (2018). Cryo-EM and X-ray structures of TRPV4 reveal insight into ion permeation and gating mechanisms. Nat. Struct. Mol. Biol..

[20] Osterwalder T, Yoon KS, White BH, Keshishian H (2001). A conditional tissue-specific transgene expression system using inducible GAL4. Proc. Natl Acad. Sci..

[21] Reilly MM, Murphy SM, Laurá M (2011). Charcot-Marie-Tooth disease. J. Peripher. Nerv. Syst..

[22] Grueber WB, Jan LY, Jan YN (2002). Tiling of the Drosophila epidermis by multidendritic sensory neurons. Development.

[23] Chen T-W (2013). Ultrasensitive fluorescent proteins for imaging neuronal activity. Nature.

[24] Niehues S (2015). Impaired protein translation in Drosophila models for Charcot–Marie–Tooth neuropathy caused by mutant tRNA synthetases. Nat. Commun..

[25] Tabuchi M (2018). Clock-generated temporal codes determine synaptic plasticity to control sleep. Cell.

[26] Thorneloe KS (2012). An orally active TRPV4 channel blocker prevents and resolves pulmonary edema induced by heart failure. Sci. Transl. Med..

[27] Hopkins AL, Groom CR (2002). The druggable genome. Nat. Rev. Drug Discov..

[28] Hell JW (2014). CaMKII: claiming center stage in postsynaptic function and organization. Neuron.

[29] Wang J-W, Beck ES, McCabe BD (2012). A modular toolset for recombination transgenesis and neurogenetic analysis of Drosophila. PLoS ONE.

[30] Park D, Coleman MJ, Hodge JJL, Budnik V, Griffith LC (2002). Regulation of neuronal excitability in Drosophila by constitutively active CaMKII. J. Neurobiol..

[31] Linley JE (2013). Perforated whole-cell patch-clamp recording. Methods Mol. Biol..

[32] Thorneloe KS (2008). N-((1S)-1-{[4-((2S)-2-{[(2,4-dichlorophenyl)sulfonyl]amino}-3-hydroxypropanoyl)-1-piperazinyl]carbonyl}-3-methylbutyl)-1-benzothiophene-2-carboxamide (GSK1016790A), a novel and potent transient receptor potential vanilloid 4 channel agonist induces urinary bladder contraction and hyperactivity: Part I.. J. Pharmacol. Exp. Ther..

[33] Willette RN (2008). Systemic activation of the transient receptor potential vanilloid subtype 4 channel causes endothelial failure and circulatory collapse: Part 2. J. Pharmacol. Exp. Ther..

[34] Sumi M (1991). The newly synthesized selective Ca2+/calmodulin dependent protein kinase II inhibitor KN-93 reduces dopamine contents in PC12h cells. Biochem. Biophys. Res. Commun..

[35] Wong MH (2019). The KN-93 molecule inhibits calcium/calmodulin-dependent protein kinase II (CaMKII) activity by binding to Ca2+/CaM. J. Mol. Biol..

[36] Ishida A, Kameshita I, Okuno S, Kitani T, Fujisawa H (1995). A novel highly specific and potent inhibitor of calmodulin-dependent protein kinase II. Biochem. Biophys. Res. Commun..

[37] Chen W, Mi R, Haughey N, Oz M, Höke A (2007). Immortalization and characterization of a nociceptive dorsal root ganglion sensory neuronal line. J. Peripher. Nerv. Syst..

[38] Wang X, Schwarz TL (2009). The mechanism of Ca2+-dependent regulation of kinesin-mediated mitochondrial motility. Cell.

[39] Guo X (2005). The GTPase dMiro is required for axonal transport of mitochondria to drosophila synapses. Neuron.

[40] Russo GJ (2009). Drosophila miro is required for both anterograde and retrograde axonal mitochondrial transport. J. Neurosci..

[41] Glater EE, Megeath LJ, Stowers RS, Schwarz TL (2006). Axonal transport of mitochondria requires milton to recruit kinesin heavy chain and is light chain independent. J. Cell Biol..

[42] Saotome M (2008). Bidirectional Ca2+-dependent control of mitochondrial dynamics by the Miro GTPase. Proc. Natl Acad. Sci..

[43] Babic M (2015). Miro’s N-terminal GTPase domain is required for transport of mitochondria into axons and dendrites. J. Neurosci..

[44] Suzuki M, Mizuno A, Kodaira K, Imai M (2003). Impaired pressure sensation in mice lacking TRPV4. J. Biol. Chem..

[45] Mizuno A, Matsumoto N, Imai M, Suzuki M (2003). Impaired osmotic sensation in mice lacking TRPV4. JAm. J. Physiol. Physiol..

[46] Materazzi S (2012). TRPA1 and TRPV4 mediate paclitaxel-induced peripheral neuropathy in mice via a glutathione-sensitive mechanism. Pflügers Arch.–Eur. J. Physiol..

[47] Boehmerle W, Huehnchen P, Lee SLL, Harms C, Endres M (2018). TRPV4 inhibition prevents paclitaxel-induced neurotoxicity in preclinical models. Exp. Neurol..

[48] McVicker DP, Millette MM, Dent EW (2015). Signaling to the microtubule cytoskeleton: an unconventional role for CaMKII. Dev. Neurobiol..

[49] Kristensen AS (2011). Mechanism of Ca2+/calmodulin-dependent kinase II regulation of AMPA receptor gating. Nat. Neurosci..

[50] Doñate-Macián P (2018). The TRPV4 channel links calcium influx to DDX3X activity and viral infectivity. Nat. Commun..

[51] Goswami C, Kuhn J, Heppenstall PA, Hucho T (2010). Importance of non-selective cation channel TRPv4 interaction with cytoskeleton and their reciprocal regulations in cultured cells. PLoS ONE.

[52] Li L (2013). Activation of transient receptor potential vanilloid 4 increases NMDA-activated current in hippocampal pyramidal neurons. Front. Cell Neurosci..

[53] Qi M (2018). Transient receptor potential vanilloid 4 activation-induced increase in glycine-activated current in mouse hippocampal pyramidal neurons. Cell Physiol. Biochem..

[54] Hong Z (2016). Transient receptor potential vanilloid 4-induced modulation of voltage-gated sodium channels in hippocampal neurons. Mol. Neurobiol..

[55] Li Y, Hu H, Tian J-B, Zhu MX, O’Neil RG (2017). Dynamic coupling between TRPV4 and Ca2+-activated SK1/3 and IK1 K+ channels plays a critical role in regulating the K+-secretory BK channel in kidney collecting duct cells. Am. J. Physiol. Ren. Physiol..

[56] Sonkusare SK (2012). Elementary Ca2+ signals through endothelial TRPV4 channels regulate vascular function. Science.

[57] Rahman M, Sun R, Mukherjee S, Nilius B, Janssen LJ (2018). TRPV4 stimulation releases ATP via pannexin channels in human pulmonary fibroblasts. Am. J. Respir. Cell Mol. Biol..

[58] Ryskamp DA (2014). Swelling and eicosanoid metabolites differentially gate TRPV4 channels in retinal neurons and glia. J. Neurosci..

[59] Heathcote HR (2019). Endothelial TRPV4 channels modulate vascular tone by Ca2+ -induced Ca2+ release at inositol 1,4,5-trisphosphate receptors. Br. J. Pharmacol..

[60] Earley S, Heppner TJ, Nelson MT, Brayden JE (2005). TRPV4 forms a novel Ca^2+^ signaling complex with ryanodine receptors and BK _Ca_ channels. Circ. Res..

[61] Camors E, Valdivia HH (2014). CaMKII regulation of cardiac ryanodine receptors and inositol triphosphate receptors. Front. Pharm..

[62] Villegas R (2014). Calcium release from intra-axonal endoplasmic reticulum leads to axon degeneration through mitochondrial dysfunction. J. Neurosci..

[63] Hou ST (2009). CaMKII phosphorylates collapsin response mediator protein 2 and modulates axonal damage during glutamate excitotoxicity. J. Neurochem.

[64] Song Y (2019). The mechanosensitive ion channel piezo inhibits axon regeneration. Neuron.

[65] Nguyen TT (2014). Loss of Miro1-directed mitochondrial movement results in a novel murine model for neuron disease. Proc. Natl Acad. Sci. USA.

[66] Zhou Y (2019). Restoring mitofusin balance prevents axonal degeneration in a Charcot-Marie-Tooth type 2A model. J. Clin. Invest.

[67] Rocha AG (2018). MFN2 agonists reverse mitochondrial defects in preclinical models of Charcot-Marie-Tooth disease type 2A. Science.

[68] Wong YC, Peng W, Krainc D (2019). Lysosomal regulation of inter-mitochondrial contact fate and motility in Charcot-Marie-Tooth Type 2. Dev. Cell.

[69] Malin SA, Davis BM, Molliver DC (2007). Production of dissociated sensory neuron cultures and considerations for their use in studying neuronal function and plasticity. Nat. Protoc..

[70] Devireddy S, Sung H, Liao P-C, Garland-Kuntz E, Hollenbeck PJ (2014). Analysis of mitochondrial traffic in drosophila. Methods Enzymol..

