# Supplementary material for: TRPV4 disrupts mitochondrial transport and causes axonal degeneration via a CaMKII-dependent elevation of intracellular Ca2+
Source: Nat Commun. 2020 May 29;11:2679. doi: 10.1038/s41467-020-16411-5 (PMC7260201; doi:10.1038/s41467-020-16411-5)
Supplement: Supplementary file 1 — Supplementary Information [file 41467_2020_16411_MOESM1_ESM.pdf]

Supplementary Materials for Woolums et al.

TRPV4 disrupts mitochondrial transport and causes axonal degeneration via a CaMKII-dependent elevation of intracellular Ca^{2+}

*Correspondence to: csumner1@jhmi.edu and tlloyd4@jhmi.edu

Supplementary Figures 1 through 13

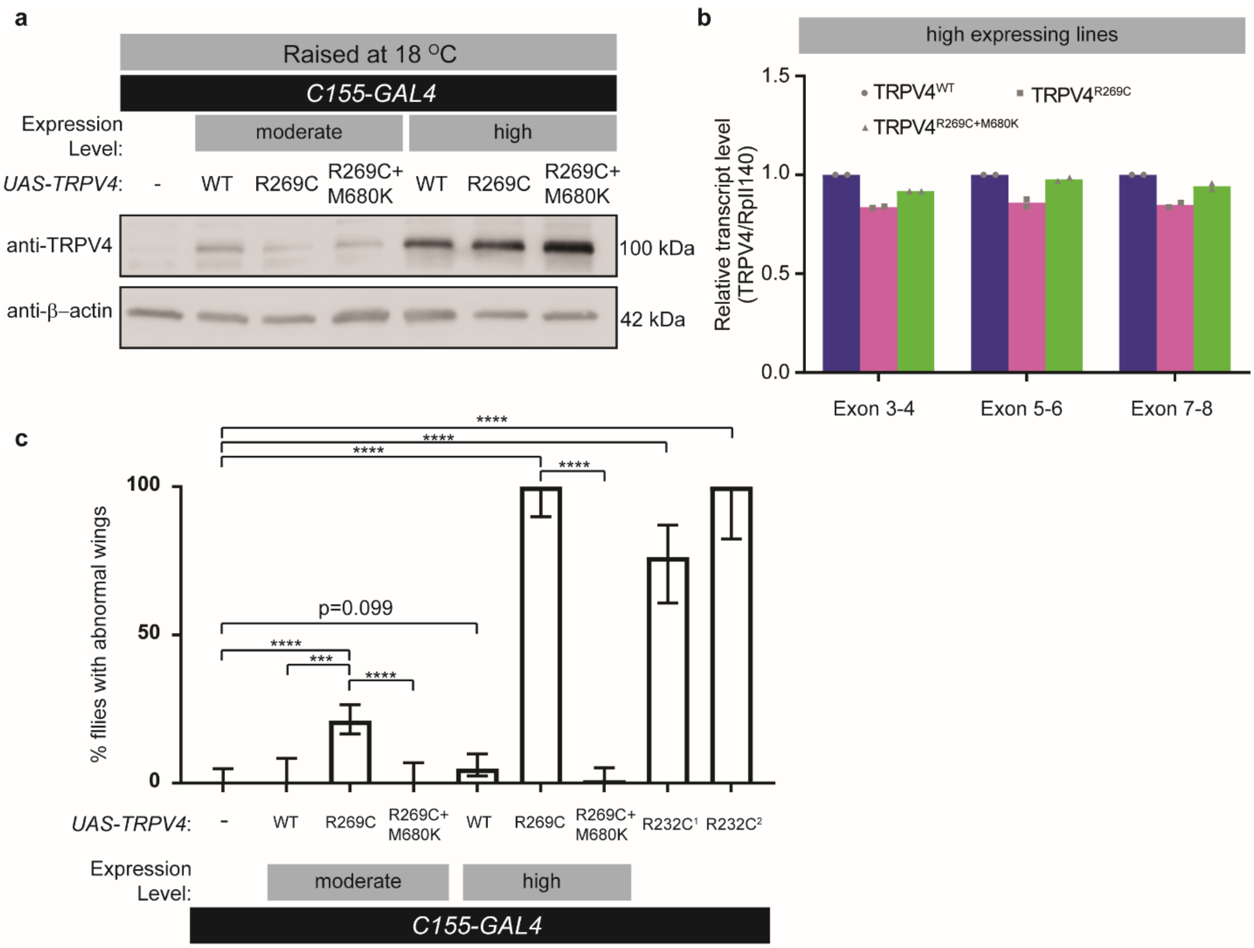

Supplementary Figure 1. Multiple neuropathy-associated TRPV4 mutant lines induce neurotoxicity. (a) Western blots of TRPV4 and β-actin from *Drosophila* head lysates prepared from flies expressing indicated TRPV4 variants raised at 18°C. Similar results observed across n=2 biological replicates. **(b)** RT-qPCR quantification of relative TRPV4 transcript from high expressing lines. Mean ± SEM. n=2 per genotype. **(c)** Percentage ± 95% CI of flies with unexpanded wings from the lines in the western blot shown in A as well as two additional transgenic lines carrying the neuropathy-associated R232C mutation. From left to right n=75, 42, 265, 52, 141, 34,105, 29, and 18 flies. X^2-test ($p<0.0001$) followed by pairwise two sided Fisher's exact test. For all panels: ***=p<0.001, and ****=p<0.0001

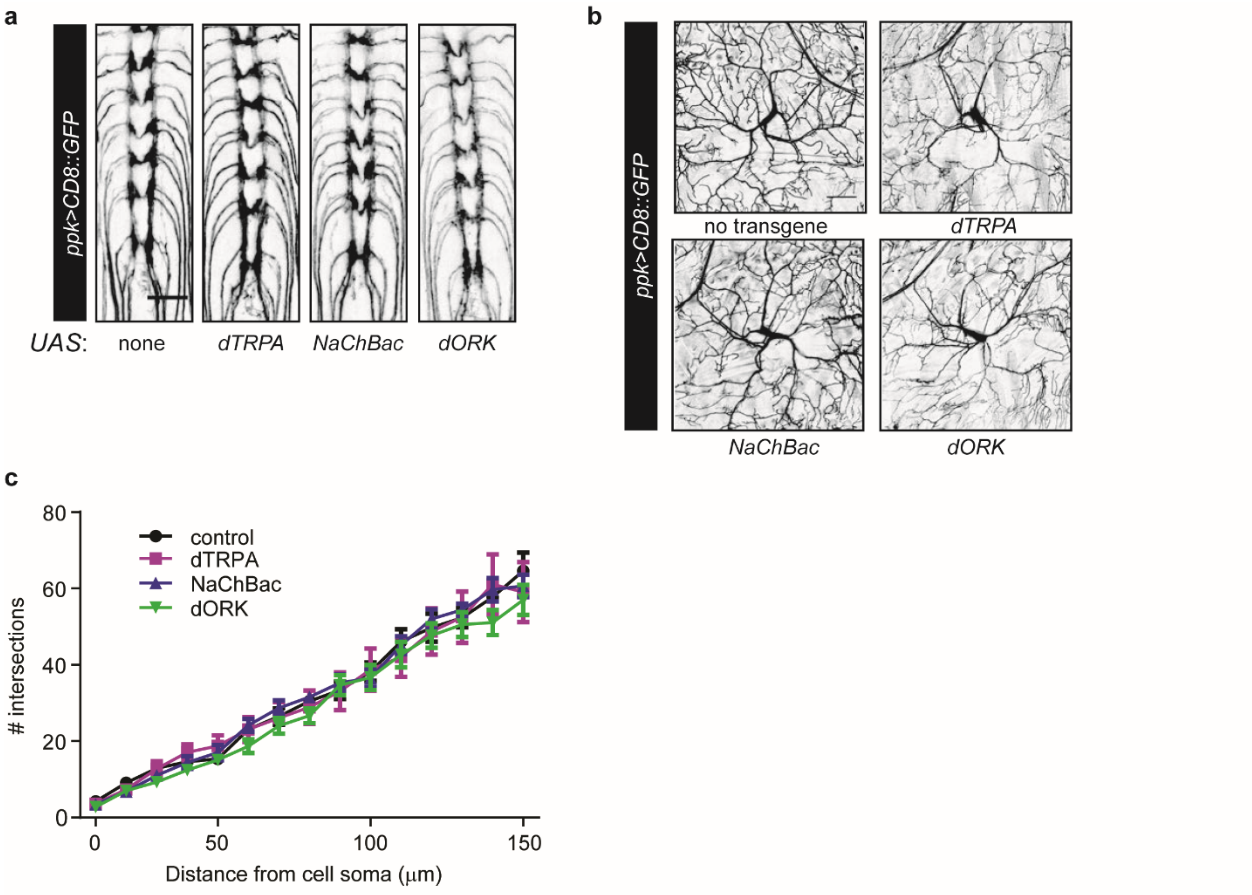

Supplementary Figure 2. Overexpression of known excitability modifiers does not cause axonal or dendritic degeneration. Confocal stacks of axonal projections **(a)** and dendrites **(b)** in C4da neurons overexpressing *Drosophila* TRPA (dTRPA), a bacterial sodium channel (NaChBac), or *Drosophila* Open Rectifier Potassium channel (dORK). Scale bar, 25 μm in **a** and 50μm in **b**. **(c)** Sholl analysis of neurons in F. n= 8 (control), 8 (dTRPA), 7 (NaChBac), and 9 (dORK) Two-way ANOVA (p>0.99).

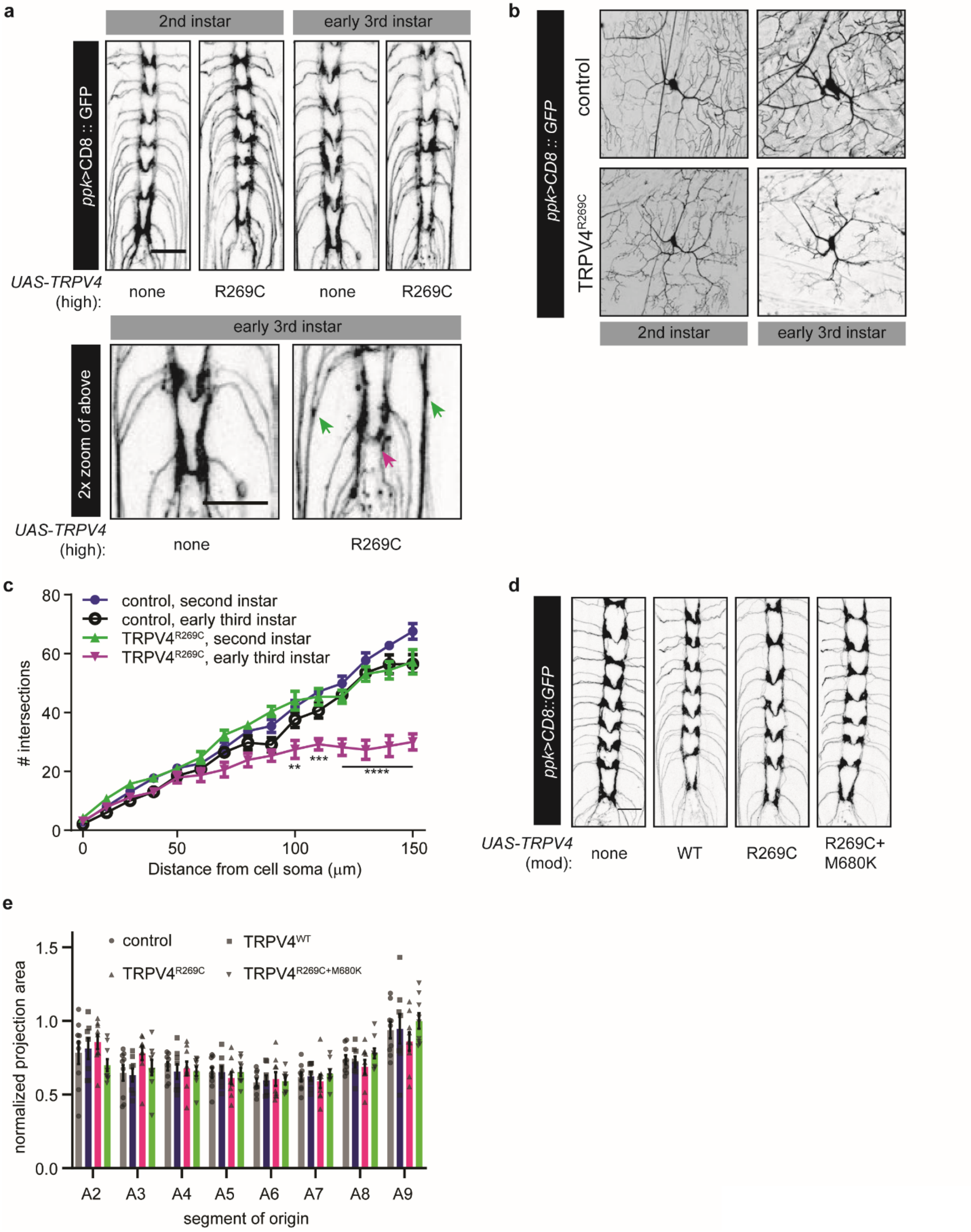

Supplementary Figure 3. Morphological phenotypes mediated by TRPV4^{R269C}(high) manifest late in development and are not phenocopied by TRPV4^{R269C}(mod). Confocal

projections of **(a)** axonal projections and **(b)** dendrites in second instar and early third instar larvae expressing no TRPV4 or TRPV4^{R269C}(high). 2x zoom in A shows a magnified image of posterior projections from the third instar control and TRPV4^{R269C} larvae above. Green arrows denote axonal swellings. Magenta arrow denotes fragmentation of projections. **(c)** Sholl analysis of the neurons in **b**. Mean ± SEM. For no TRPV4 second instar and third instar n= 9 and 8, respectively. For TRPV4^{R269C}, n= 8 per time point. Two-way ANOVA (p<0.0001), Tukey's *post hoc* test. Asterisks indicate comparison of third instar no TRPV4 larvae to third instar TRPV4^{R269C} larvae. **(d)** Confocal projections of C4da neuron axonal projections in larvae expressing TRPV4(mod) variants. **(e)** Quantification of normalized projection area in D. Mean ± SEM. n= 9 (no TRPV4),8 (TRPV4^{WT}),9 (TRPV4^{R269C}),9 (TRPV4$^{R269C+M680K}$). Two-way ANOVA (p=0.48). Scale bar, 25 μm in A, D, and G, 50 μm in B and E. For all panels: **=p<0.01, ***=p<0.001, and ****=p<0.0001

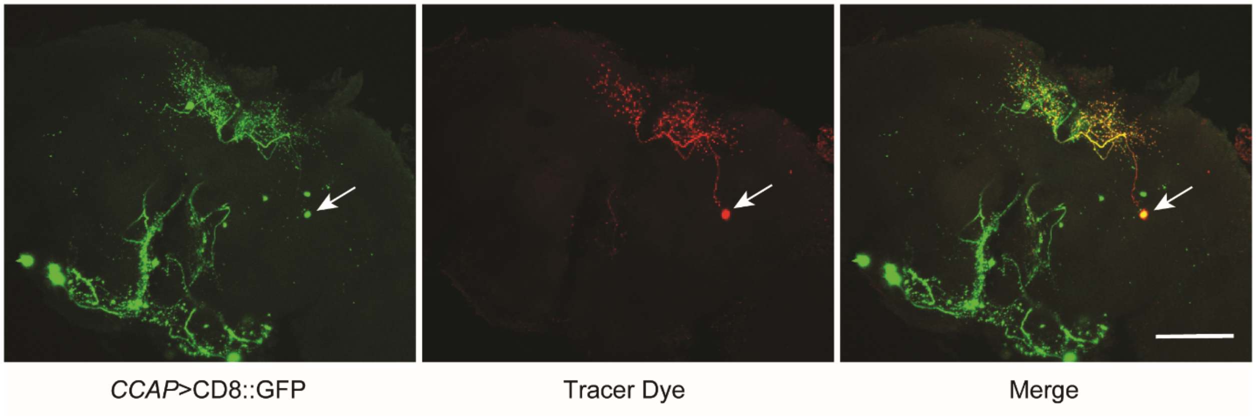

Supplementary Figure 4. Confirmation of recording from N_{CCAP}. Confocal projections of N_{CCAP} labeled by CD8::GFP, tracer dye (red, injected after recording), and their colocalization. White arrow indicates CD8::GFP positive cell body co-localized with the tracer dye. Similar results observed across n=5 *Drosophila* brains. Scale bar, 100 μm.

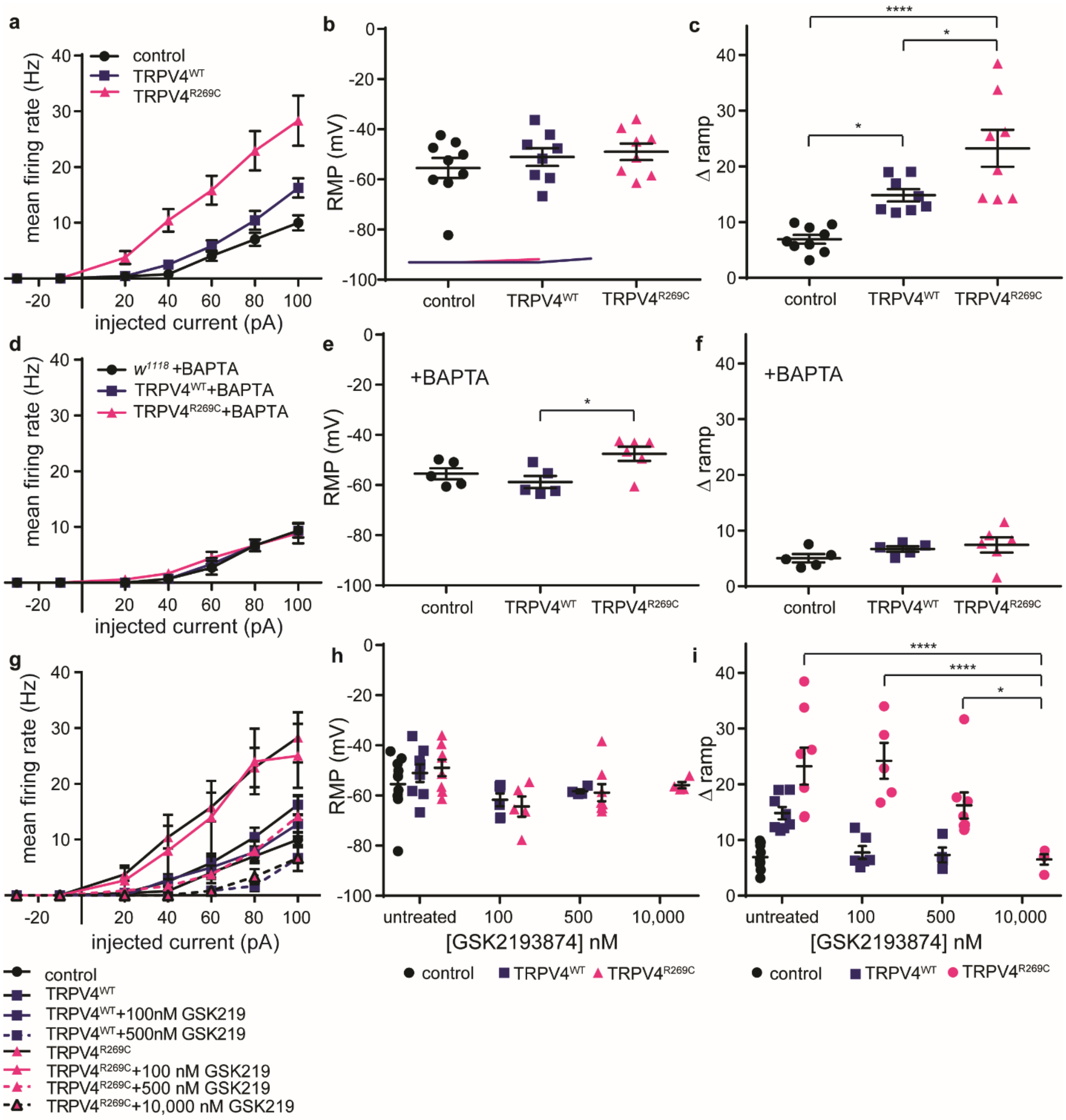

Supplementary Figure 5. TRPV4^{R269C} activity increases neuronal excitability in a Ca^{2+}-dependent manner. Evoked mean firing rate in response to inject current **(a),** resting membrane potential (RMP) **(b),** and membrane potential variability (Δ ramp) **(c)** in flies of the indicated genotypes. Mean ± SEM. For B-D, n = 9 (no TRPV4), 8 (TRPV4^{WT}), and 8 (TRPV4^{R269C}). **a**: Two-way ANOVA (p<0.0001), Tukey's *post hoc* test. Asterisks indicate

difference from control. **b**: One-way ANOVA ($p=0.45$). **c**: One-way ANOVA($p<0.0001$), Tukey's *post hoc* test. Evoked firing rate **(d),** RMP **(e)** and Δ ramp **(f)** in flies of the indicated genotypes when treated with 5 mM BAPTA. Mean ± SEM. For **d-f**, n= 5 (no TRPV4), 5 ($TRPV4^{WT}$) and 6 ($TRPV4^{R269C}$). **d**:Two-way ANOVA($p=0.99$). **e**: One-way ANOVA($p=0.021$), Tukey's *post hoc* test. **f**: One-way ANOVA($p=0.27$). Evoked mean firing rate **(g),** RMP **(h),** and Δ ramp **(i)** after bath application of GSK219 for 1 hour at the indicated concentrations. Mean ± SEM. For **g-i**, $TRPV4^{WT}$ n = 8, 6 and 4 for 0, 100, and 500 nM. $TRPV4^{R269C}$ = 8, 5, 8, and 4 for 0, 100, 500 and 10,000 nM. **h**: One-way ANOVA, $TRPV4^{WT}$ ($p=0.059$), $TRPV4^{R269C}$ ($p<0.031$), Tukey's *post hoc* test. **i**: One-way ANOVA, $TRPV4^{WT}$ ($p=0.0004$), $TRPV4^{R269C}$ ($p=0.0044$), Tukey's *post hoc* test. For all panels: *=p<0.05, **=p<0.01, ***=p<0.001, ****=p<0.0001.

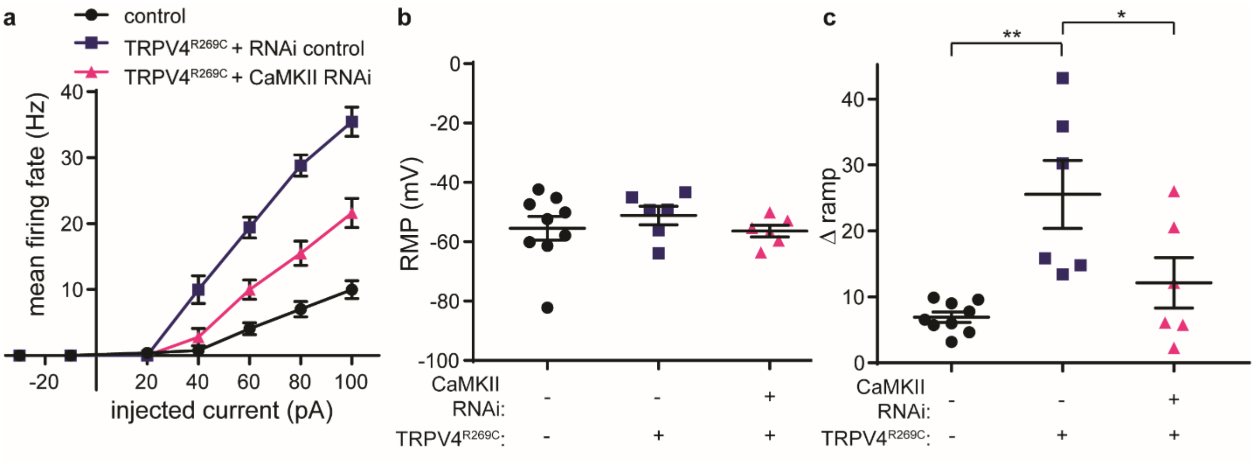

Supplementary Figure 6. TRPV4^{R269C} activity increases neuronal excitability in a CaMKII-dependent manner. Evoked mean firing rate **(a),** RMP **(b),** and Δ ramp **(c)** in flies of the indicated genotypes. Mean ± SEM, n =8, 6, and 6**. a:** Two-way ANOVA (p<0.0001), Tukey's *post hoc* test, asterisks indicate difference from TRPV4^{R269C}+RNAi control. **b**: One-way ANOVA (p=0.58). **c**: One-way ANOVA (p=0.0004), Tukey's *post hoc* test. For all panels: *=p<0.05, **=p<0.01, ***=p<0.001, ****=p<0.0001.

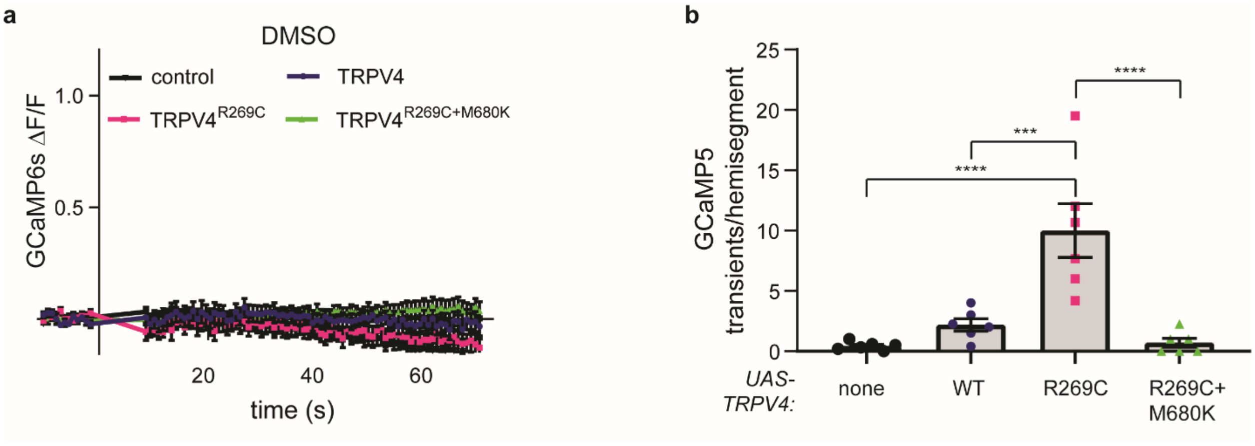

Supplementary Figure 7. Intracellular Ca^{2+} in C4da neurons expressing TRPV4 variants.

(a) GCaMP6S ΔF/F over time in C4da neurons expressing TRPV4 variants upon DMSO addition at t=0s. DMSO does not increase intracellular calcium. **(b)** Quantitation of spontaneous GCaMP5 signals in larval ventral nerve cord of animals expressing TRPV4(mod). Calcium transients per hemisegment per minute were counted (at least 3 hemisegments per animal). Mean ± SEM. n = 6 per genotype. One-way ANOVA ($p < 0.0001$), Tukey's *post hoc* test. For all panels: ***=$p<0.001$, ****=$p<0.0001$.

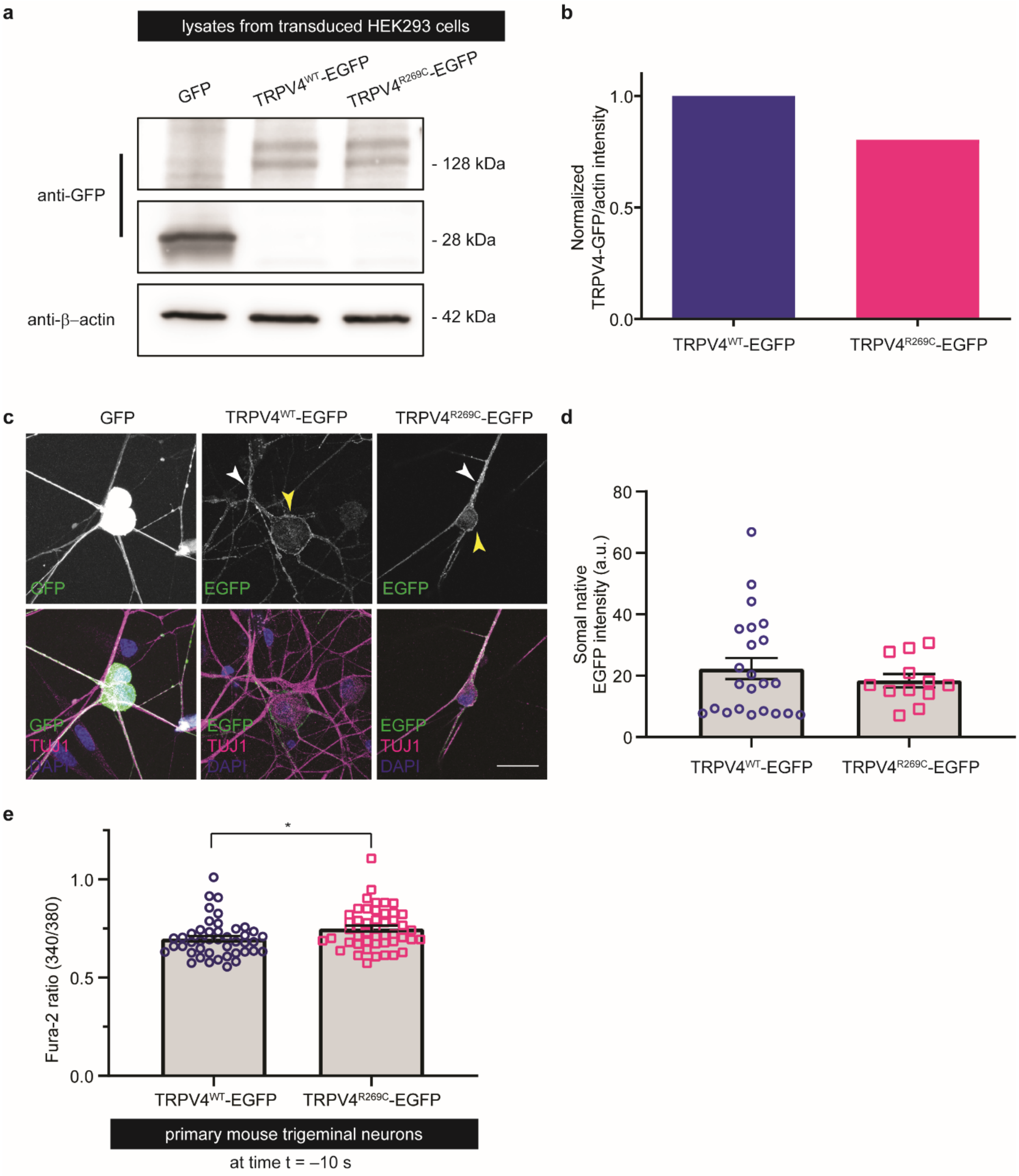

Supplementary Figure 8. TRPV4 localizes to the cell cortex of primary neurons and TRPV4^{R269C} slightly elevates basal calcium. (a) Western blots of GFP and β-actin from HEK293T cell lysates prepared from cells transduced with GFP, TRPV4^{WT}-EGFP or TRPV4^{R269C}-EGFP**. (b)** Ratio of anti-GFP to anti-β-actin signal in **a. (c)** Confocal images of

primary trigeminal neurons transduced with lentivirus to express GFP, $TRPV4^{WT}$-EGFP, or $TRPV4^{R269C}$-EGFP. Yellow arrows: $TRPV4^{WT}$-EGFP membrane enrichment, white arrow: localization of $TRPV4^{WT}$-EGFP to neuronal processes. Scale bar, 25 μm. **(d)** From a separate experiment, quantification of trigeminal neuron somal native EGFP intensity in neurons transduced to express $TRPV4^{WT}$-EGFP or $TRPV4^{R269C}$-EGFP. Mean ± SEM. n = 23 and 12 cells for $TRPV4^{WT}$-EGFP and $TRPV4^{R269C}$-EGFP, respectively. Unpaired two-tailed t-test (p = 0.42). **(e)** Quantification of Fura-2AM 340/380 ratio at t = -10 s from **Fig.5d**. Mean ± SEM. n = 44 and 48 cells for $TRPV4^{WT}$-EGFP and $TRPV4^{R269C}$-EGFP, respectively. Unpaired two-tailed t-test. *p=0.0146.

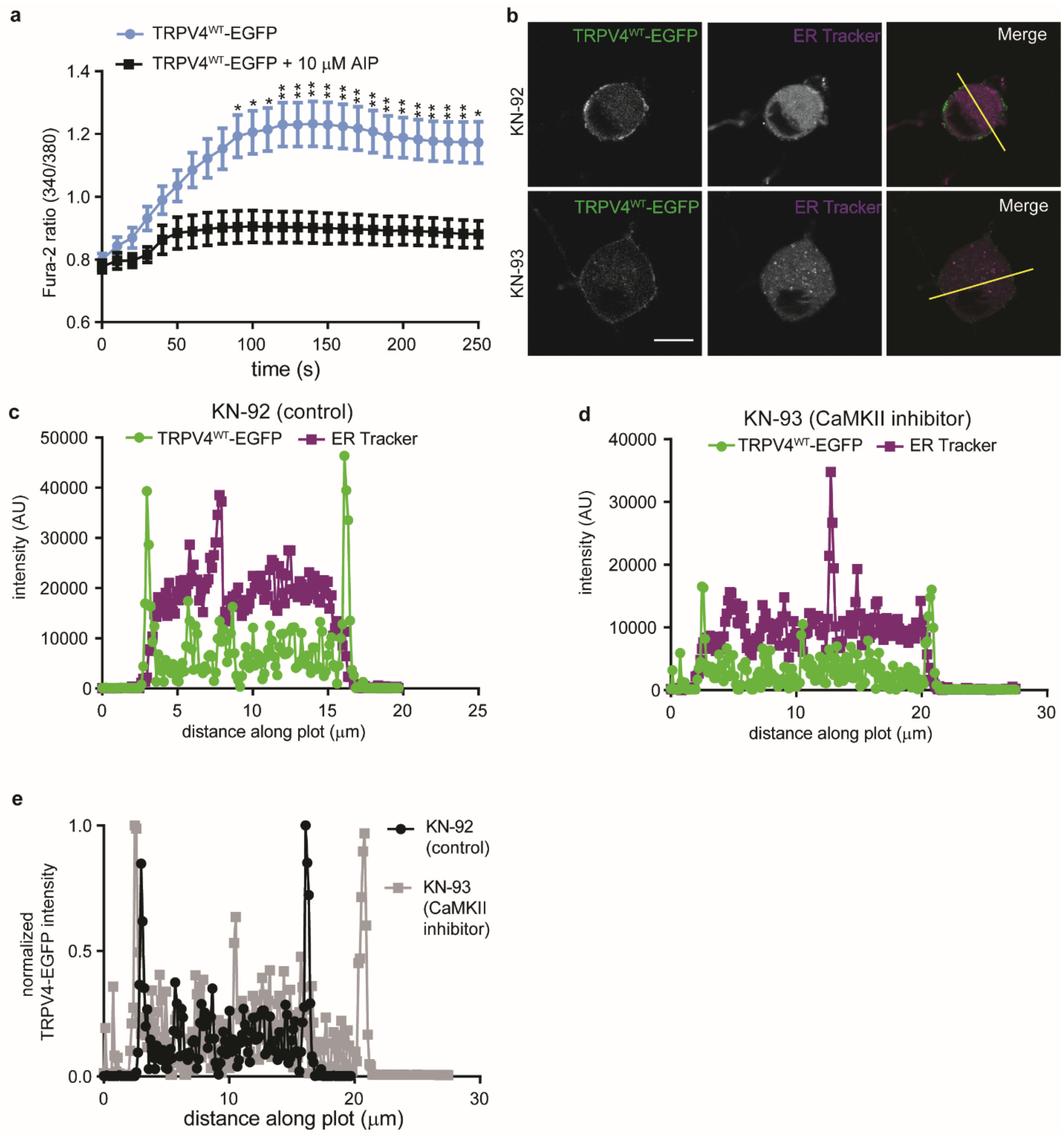

Supplementary Figure 9. CaMKII does not alter TRPV4 localization to the cell cortex.

(a) Mean ± SEM Fura-2AM ratio over time in neurons transduced to express TRPV4-EGFP and treated with either vehicle or 10 μM autocamtide-2-related inhibitor peptide (AIP) followed by treatment with 30 nM GSK101 at time=0. n= 46 (control) and 37 (AIP) neurons. Two-way ANOVA, Geisser-Greenhouse correction ($p<0.0001$), Tukey's *post hoc* test. *=$p<0.05$, **=$p<0.0$,

***=$p<0.001$, ****=$p<0.0001$ **(b)** Confocal images of primary trigeminal neurons transduced to express TRPV4^{WT}-EGFP treated with either 10 μM KN-92 or KN-93. Yellow lines indicate the length along which the line scans in panels **c, d and e** were derived. Scale bar, 25 μm. **(c)** Intensity profile of ER-Tracker Red and EGFP in a neuron transduced with TRPV4^{WT}-EGFP and treated with 10 μM KN-92. **(d)** Intensity profile of ER-Tracker Red and EGFP in a neuron transduced with TRPV4^{WT}-EGFP and treated with 10 μM KN-93. **(e)** Superimposed normalized traces from **c** and **d**. Values were normalized to the maximum value within either trace.

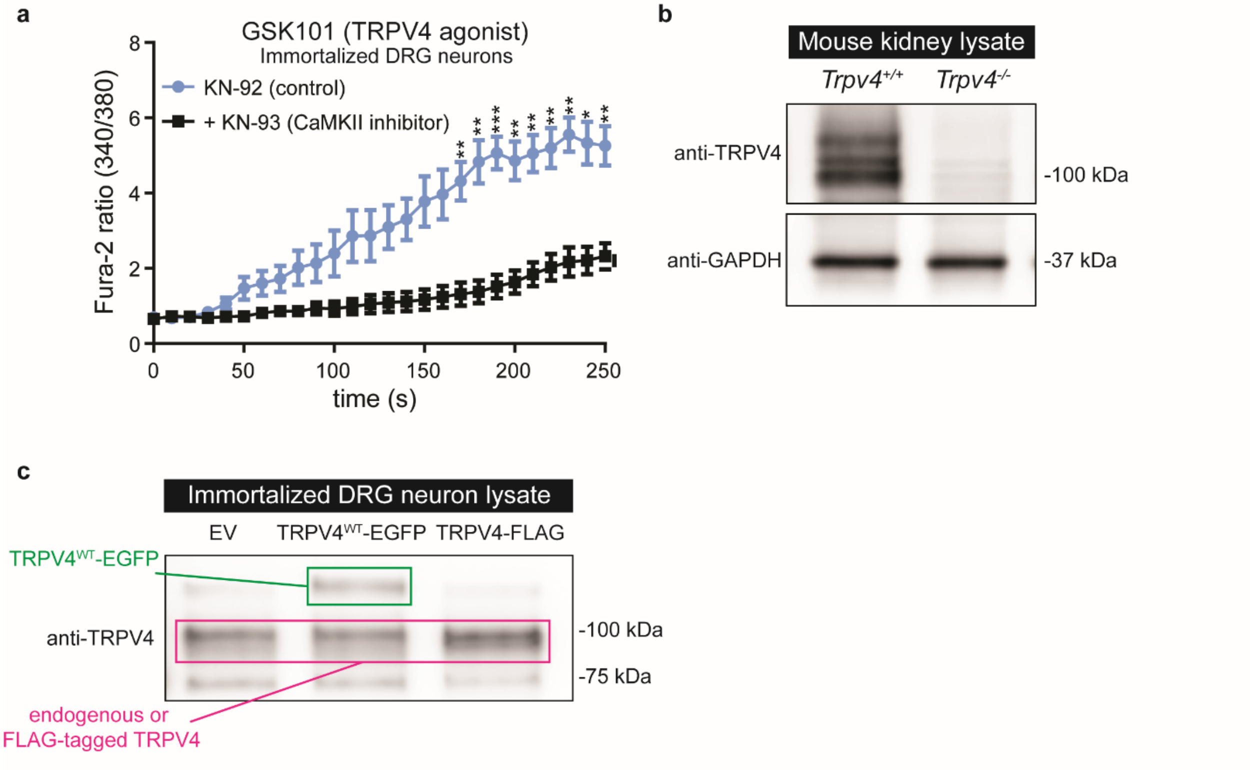

Supplementary Figure 10. CaMKII inhibition prevents increases in intracellular Ca^{2+} mediated by endogenous TRPV4. (a) Mean ± SEM Fura-2AM ratio over time in immortalized rat DRG neurons (50B11 cells) that have been treated for 4 hours with either 10 µM KN-92 or 10 µM KN-93 followed by stimulation with 30 nM GSK101 at time=0. n=9 coverslips of cells per condition. Two-way ANOVA, Geisser-Greenhouse correction (p<0.0001), Tukey's *post hoc* test. **(b)** Western blot of mouse kidney lysate from wild type and *Trpv4*$^{-/-}$ mice demonstrates antibody specificity. Similar results were observed in 3 separate experiments. **(c)** Western blot of 50B11 lysates probed with the same antibody as I. Cells were transfected with empty vector (EV), TRPV4-EGFP, or TRPV4-FLAG. Similar results were observed in 3 separate experiments. For all panels: *=p<0.05, **=p<0.0, ***=p<0.001, ****=p<0.0001

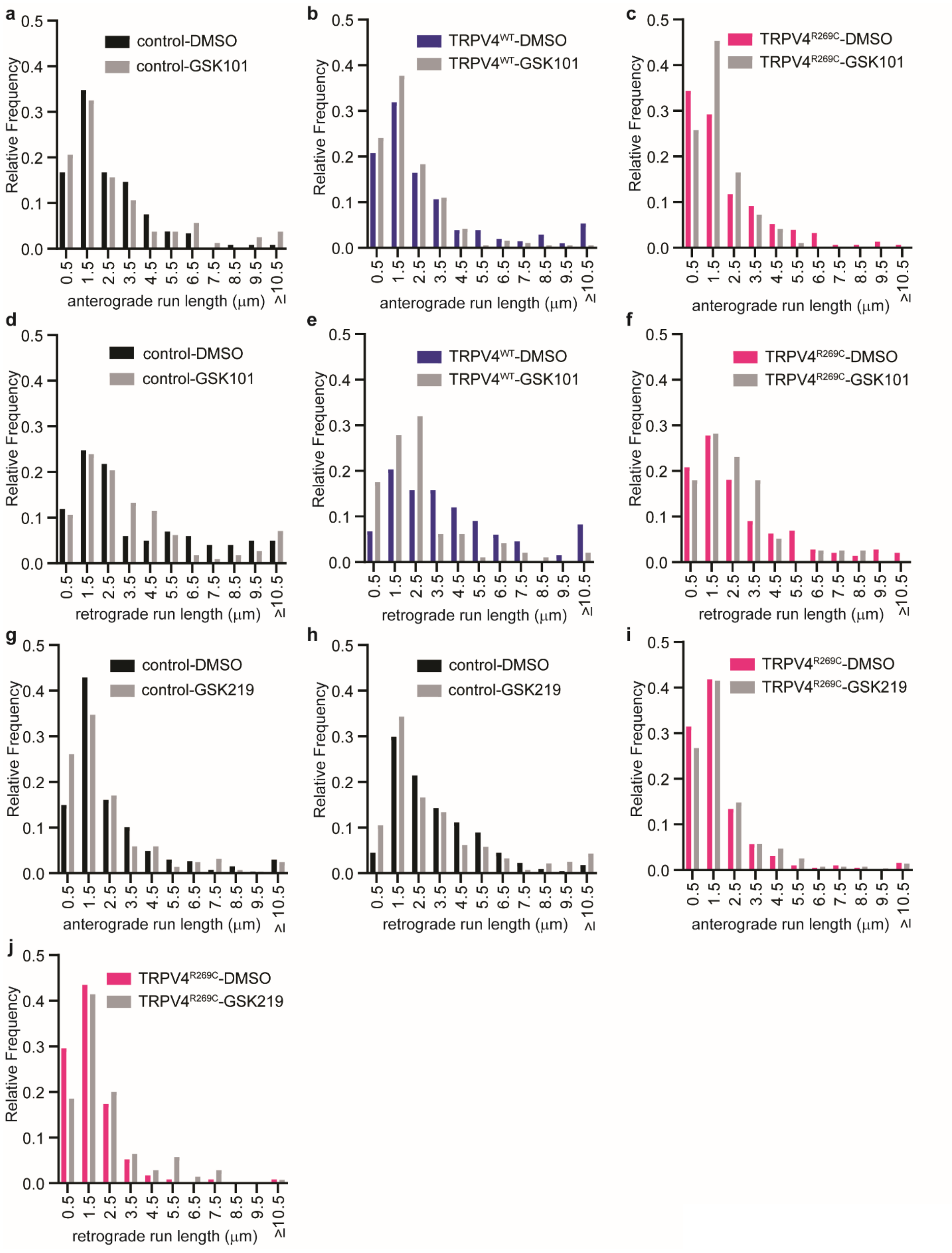

Supplementary Figure 11. TRPV4 activation causes a modest shift to shorter mitochondrial run lengths. Histogram of anterograde run lengths in larvae treated with DMSO

or 40 nM GSK101 and expressing **(a)** no TRPV4 (n= 239 (DMSO), 160 (GSK101) runs), **(b)** $TRPV4^{WT}$ (n= 207, 191), or **(c)** $TRPV4^{R269C}$ (n=154, 97). Histogram of retrograde run lengths in larvae treated with DMSO or 40 nM GSK101 and expressing **(d)** no TRPV4 (n=101, 113), **(e)** TRPV4 (n=133, 97), or **(f)** $TRPV4^{R269C}$(n=144, 39). (G-H) Histograms of mitochondrial run lengths in larvae treated with DMSO or 10 μM GSK219 and expressing no TRPV4 in the **(g)** anterograde (n= 268 (DMSO), 288 (GSK219) runs) and **(h)** retrograde (n= 224, 277) directions. Histograms of mitochondrial run lengths in larvae treated with DMSO or 10 μM GSK219 and expressing $TRPV4^{R269C}$ in the **(i)** anterograde (n= 194, 277) and **(j)** retrograde (n= 115, 140) directions.

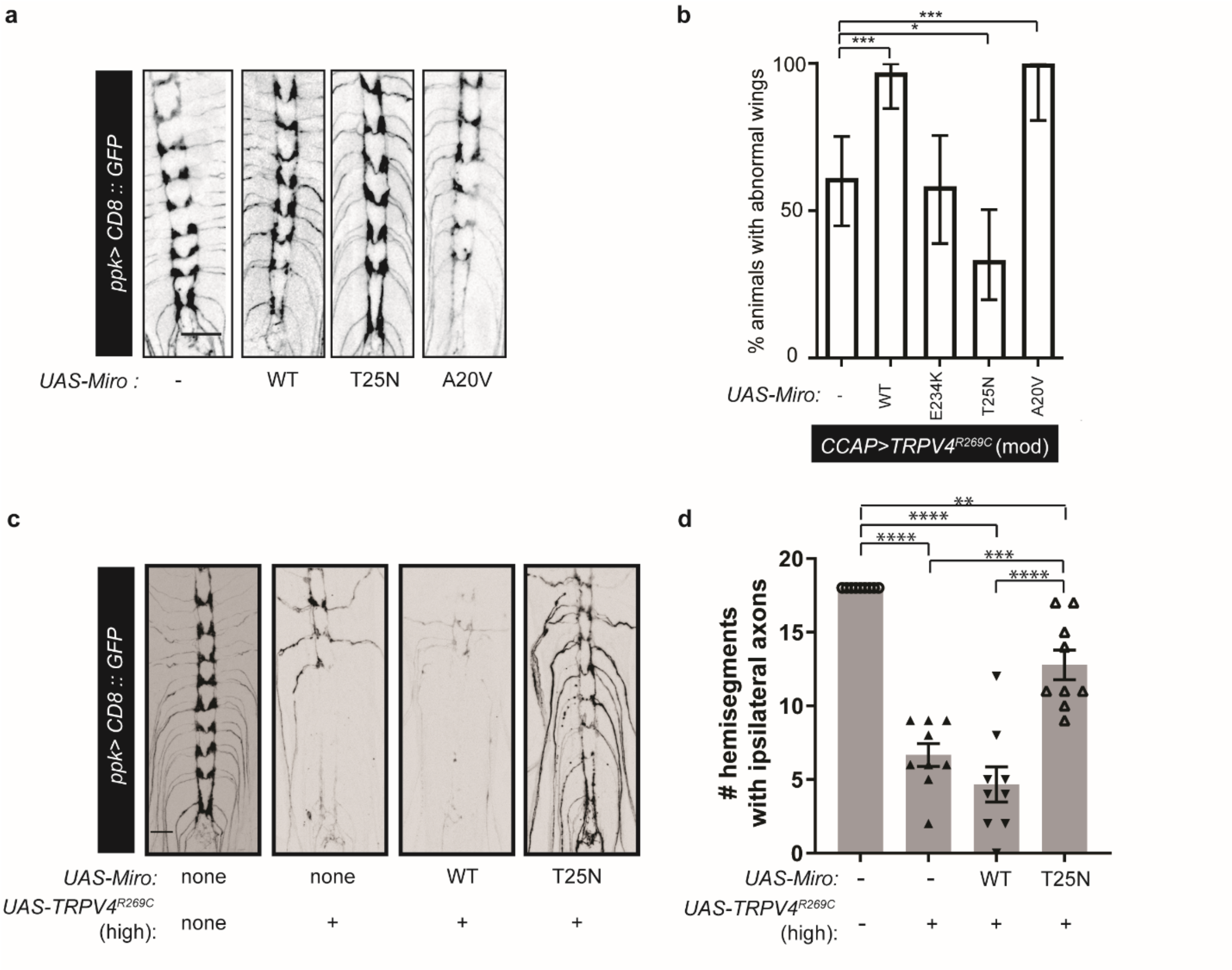

Supplementary Figure 12. Wild type Miro overexpression does not cause observable axonal degeneration in isolation and overexpression of GDP-bound Miro mildly suppresses TRPV4^{R269C}-mediated toxicity. **(a)** Confocal projections of C4da axonal projections in larvae overexpressing wild type Miro, GDP-bound Miro (Miro^{T25N}), or GTP-bound Miro (Miro^{A20V}). **(b)** Percentage ± 95% CI of flies with unexpanded wings when co-expressing TRPV4^{R269C}(mod) with Miro variants. From left to right n= 36, 33, 24, 33, and16 flies. X^2-test ($p<0.0001$) followed by pairwise two-sided Fisher's exact test. **(c)** Confocal projections of C4da axonal projections in larvae overexpressing high levels of TRPV4^{R269C} and either wild type Miro or Miro^{T25N}. **(d)** Innervation of ipsilateral synaptic hemisegments shown in C. n= 9 larvae per genotype. Mean ± SEM. One-way ANOVA($p<0.0001$), Tukey's *post hoc* test Scale bar, 25 μM in B and C. For all panels: **=p<0.01, ***=p<0.001, ****=p<0.0001

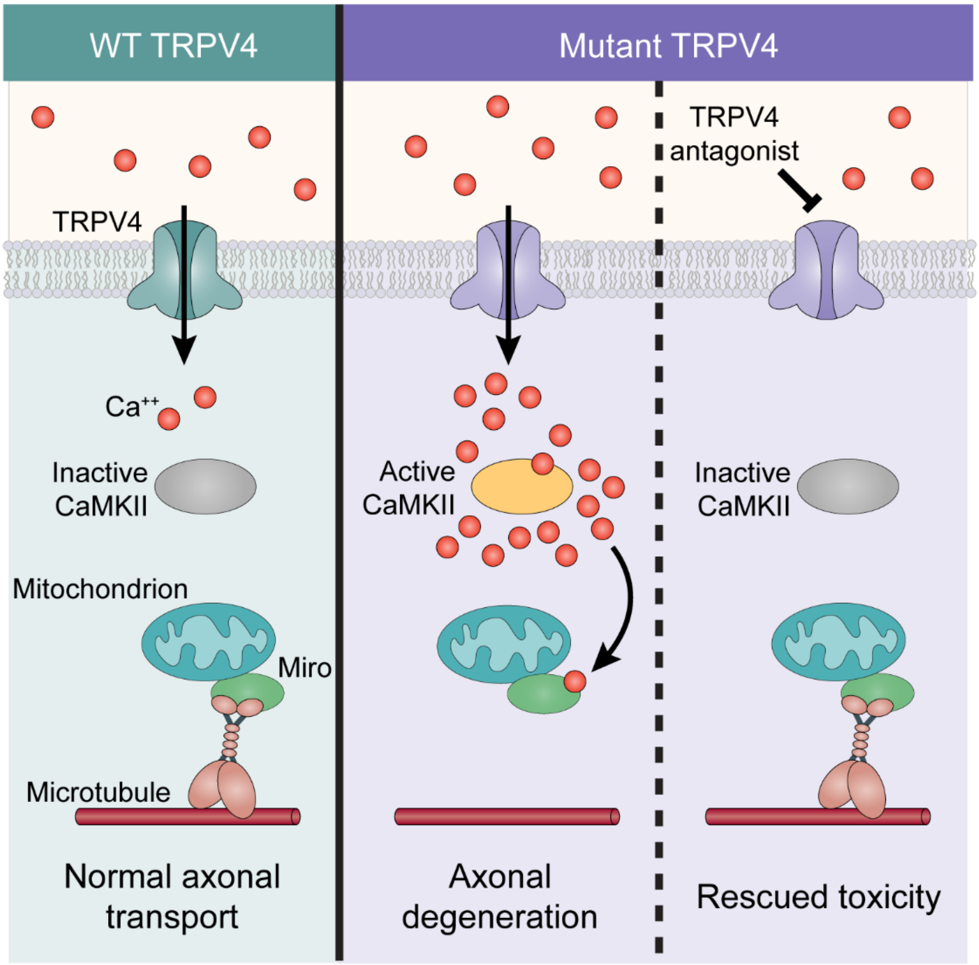

Supplementary Figure 13. Model of TRPV4-mediated neurotoxicity. Activation of TRPV4, which is more rapid in the case of neuropathogenic mutants, causes an increase in intracellular Ca^{2+} that is dependent on CaMKII. This increase in intracellular Ca^{2+} disrupts mitochondrial transport by increasing the stationary pool of mitochondria. Additionally, TRPV4-mediated Ca^{2+} influx causes axonal degeneration which is enhanced by the mitochondrial transport protein Miro in a manner dependent on Miro binding to Ca^{2+}. This implies a Ca^{2+}-dependent relationship between mitochondrial axon transport machinery and axonal degeneration. Genetic or pharmacologic blockade of either TRPV4 or CaMKII activity prevents TRPV4-mediated Ca^{2+} increases and strongly suppresses axonal degeneration and neuronal dysfunction.
