# Supplementary material for: TRPV4 disrupts mitochondrial transport and causes axonal degeneration via a CaMKII-dependent elevation of intracellular Ca2+
Source: Nat Commun. 2020 May 29;11:2679. doi: 10.1038/s41467-020-16411-5 (PMC7260201; doi:10.1038/s41467-020-16411-5)
Supplement: Supplementary file 9 — Reporting Summary [file 41467_2020_16411_MOESM9_ESM.pdf]

nature research

Corresponding author(s): Thomas E. Lloyd, Charlotte J. Sumner

Last updated by author(s): Apr 23, 2020

Reporting Summary

Nature Research wishes to improve the reproducibility of the work that we publish. This form provides structure for consistency and transparency in reporting. For further information on Nature Research policies, see Authors & Referees and the Editorial Policy Checklist.

Statistics

For all statistical analyses, confirm that the following items are present in the figure legend, table legend, main text, or Methods section.

n/a	Confirmed	
☐	☒	The exact sample size (*n*) for each experimental group/condition, given as a discrete number and unit of measurement
☐	☒	A statement on whether measurements were taken from distinct samples or whether the same sample was measured repeatedly
☐	☒	The statistical test(s) used AND whether they are one- or two-sided<br>*Only common tests should be described solely by name; describe more complex techniques in the Methods section.*
☒	☐	A description of all covariates tested
☐	☒	A description of any assumptions or corrections, such as tests of normality and adjustment for multiple comparisons
☐	☒	A full description of the statistical parameters including central tendency (e.g. means) or other basic estimates (e.g. regression coefficient) AND variation (e.g. standard deviation) or associated estimates of uncertainty (e.g. confidence intervals)
☐	☒	For null hypothesis testing, the test statistic (e.g. *F*, *t*, *r*) with confidence intervals, effect sizes, degrees of freedom and *P* value noted<br>*Give P values as exact values whenever suitable.*
☒	☐	For Bayesian analysis, information on the choice of priors and Markov chain Monte Carlo settings
☒	☐	For hierarchical and complex designs, identification of the appropriate level for tests and full reporting of outcomes
☒	☐	Estimates of effect sizes (e.g. Cohen's *d*, Pearson's *r*), indicating how they were calculated

Our web collection on statistics for biologists contains articles on many of the points above.

Software and code

Policy information about availability of computer code

Data collection	pCLAMP 10 software (Molecular Devices) was used to collect electrophysiological data. Zeiss Zen Blue 2.3 software was used to collect confocal imaging data. AxioVision was used to collect calcium imaging data.
Data analysis	Electrophysiological analysis was performed custom MATLAB-based software (code available from Masashi Tabuchi, Dept. of Neurosciences, Case Western Reserve University School of Medicine, 2210 Circle Dr, Cleveland, OH, 44106, USA, dorcusmasashi@gmail.com.). Imaging analysis was performed using ImageJ (NIH), Zen Blue (Zeiss) or Imaris (Bitplane, Zurich, Switzerland). Statistical analysis was performed using Prism 7.0 (GraphPad).

For manuscripts utilizing custom algorithms or software that are central to the research but not yet described in published literature, software must be made available to editors/reviewers. We strongly encourage code deposition in a community repository (e.g. GitHub). See the Nature Research guidelines for submitting code & software for further information.

Data

Policy information about availability of data

All manuscripts must include a data availability statement. This statement should provide the following information, where applicable:

- Accession codes, unique identifiers, or web links for publicly available datasets
- A list of figures that have associated raw data
- A description of any restrictions on data availability

All data supporting the findings of this study and unique biological materials used in this study are available from the corresponding authors upon reasonable request. The source data for Figures 1b, e, f; 2b, d, g; 3c, e, f, g; 4b, c, e; 5b, d, e; 6b-e; 7b, d-f and Supplementary Figures 1a-c; 2c; 3c, e; 5a-i; 6a-c; 7a, b; 8a, b, d, e; 9a, c-e; 10a-c; 11a-j; 12b, d are provided in the Source Data file.

Field-specific reporting

Please select the one below that is the best fit for your research. If you are not sure, read the appropriate sections before making your selection.

☒ Life sciences ☐ Behavioural & social sciences ☐ Ecological, evolutionary & environmental sciences

For a reference copy of the document with all sections, see nature.com/documents/nr-reporting-summary-flat.pdf

Life sciences study design

All studies must disclose on these points even when the disclosure is negative.

Sample size	sample sizes were not predetermined, sample sizes were deemed sufficient once the Standard Error of the mean was approximately 10% of the mean or if similar sample sizes had been previously reported in the literature (e.g. Drosophila climbing assays)
Data exclusions	no data were excluded
Replication	experiments were replicated at least twice and none shown failed to replicate. Live cell imaging experiments or electrophysiology experimental data were pooled over the course of 2-4 recording sessions that occurred on different days
Randomization	For live imaging experiments or physiology recordings, differing genotypes or treatment conditions were recorded in an alternating fashion (i.e. treatment 1, treatment 2, treatment 1, treatment 2). Organisms and cells were assigned to treatment groups at random. For larval dissections and climbing assays, subjects were selected at random. For wing expansion assays, all flies present in the vial were scored.
Blinding	fluorescent image quantification that were not solely based on objective fluorescent intensity thresholds were blinded by another lab member not performing the quantification changing the file names, images were quantified and then unblinded. Blinding was also perfomed during climbing assay experiments by having a lab member who was not performing the assay transfer flies to vials with blinded labels so the person performing the assay was not aware of the genotype during the assay. Blinding was not performed for other experiments.

Reporting for specific materials, systems and methods

We require information from authors about some types of materials, experimental systems and methods used in many studies. Here, indicate whether each material, system or method listed is relevant to your study. If you are not sure if a list item applies to your research, read the appropriate section before selecting a response.

Materials & experimental systems

n/a	Involved in the study	
☐	☒	Antibodies
☐	☒	Eukaryotic cell lines
☒	☐	Palaeontology
☐	☒	Animals and other organisms
☒	☐	Human research participants
☒	☐	Clinical data

Methods

n/a	Involved in the study	
☒	☐	ChIP-seq
☒	☐	Flow cytometry
☒	☐	MRI-based neuroimaging

Antibodies

Antibodies used	polyclonal rabbit anti-TRPV4 (1:500; Abcam; ab39260); monoclonal rabbit anti-β-actin (1:1000; Cell Signaling Technology; #4970); monoclonal mouse anti-GAPDH (1:5000; ThermoFisher; #AM4300); HRP-conjugated mouse anti-rabbit (1:150,000; Jackson ImmunoResearch; #211-032-171); HRP-cojugated goat anti-mouse (1:150,000; Jackson ImmunoResearch; #211-032-171); mouse anti-GFP IgG2a (1:1,000, ThermoFisher, A-11120); rabbit anti-GFP (1:1,000, ThermoFisher, A-11122); DyLight 488-conjugated goat anti-mouse IgG2a (1:1,000, Jackson ImmunoResearch, 115-285-206); Alexa Fluor 488-conjugated goat-anti rabbit IgG (1:1,000, ThermoFisher, A-11034); chicken anti-TUJ1 (1:1,000 , MilleporeSigma, AB9354)
Validation	rabbit anti-TRPV4 (Abcam; ab39260) was validated by the company (https://www.abcam.com/trpv4-antibody-ab39260.html) and on-site through the use of TRPV4 knockout mice (Supplemetary Figure 9). rabbit anti-β-actin (Cell Signaling Technology; #4970) was validated by the company (https://www.cellsignal.com/products/primary-antibodies/b-actin-13e5-rabbit-mab/4970). mouse anti-GAPDH (ThermoFisher; #AM4300) was validated by the company and is widely used in the literature (https://www.thermofisher.com/antibody/product/GAPDH-Antibody-clone-6C5-Monoclonal/AM4300). mouse anti-GFP IgG2a (ThermoFisher, A-11120) was validated by the company and is widely used in the literature (https://www.thermofisher.com/antibody/product/GFP-Tag-Antibody-clone-3E6-Monoclonal/A-11120). rabbit anti-GFP (ThermoFisher, A-11122) was validated by the company and is widely used in the literature (https://www.thermofisher.com/antibody/product/GFP-Antibody-Polyclonal/A-11122). chicken anti-TUJ1 (MilleporeSigma, AB9354) was validated by the company and is widely used in the literature (http://www.emdmillipore.com/US/en/product/Anti-Beta-III-Tubulin-Antibody,MM_NF-AB9354#overview).

Eukaryotic cell lines

Policy information about cell lines

Cell line source(s)	50B11 cells were from Ahmet Hoke
Authentication	observed differentiation with cell line upon treatment with forskolin as reported in the literature (Chen et al. Journal of Perphipheral Nervous System, 2007). Other methods of validation were not performed.
Mycoplasma contamination	cell lines were not test for mycoplasm contamination
Commonly misidentified lines (See ICLAC register)	no commonly misidentified cell lines were used

Animals and other organisms

Policy information about studies involving animals; ARRIVE guidelines recommended for reporting animal research

Laboratory animals	C57BL6J mice, male and female, aged 6-12 weeks were used for primary trigeminal neuron preparations; transgenic Drosophila, male and female, where used from 2nd instar larval stage through 15 days post-eclosion, depending on the assay.
Wild animals	Study did not involve wild animals
Field-collected samples	Study did not involve field-collected samples
Ethics oversight	All mice used in this study were housed and handled according to protocols approved by the Animal Care and Use Committee of Johns Hopkins University School of Medicine

Note that full information on the approval of the study protocol must also be provided in the manuscript.
