# Supplementary material for: TRPV4 disrupts mitochondrial transport and causes axonal degeneration via a CaMKII-dependent elevation of intracellular Ca2+
Source: Nat Commun. 2020 May 29;11:2679. doi: 10.1038/s41467-020-16411-5 (PMC7260201; doi:10.1038/s41467-020-16411-5)
Supplement: Supplementary file 2 — Peer Review File [file 41467_2020_16411_MOESM2_ESM.docx]

Peer Review Information

**Journal:** Nature Communications

**Manuscript title:** TRPV4 disrupts mitochondrial transport and causes axonal degeneration via a CaMKII-dependent elevation of intracellular Ca^2+^

**Corresponding author name(s):** Dr Thomas Lloyd

**Editorial notes:**

**none**

**Reviewer comments & decisions:**

**Reviewer comments, first version:**

Reviewers' comments:

Reviewer #1 (Remarks to the Author):

In this manuscript, Woolums et al. utilize Drosophila genetics and cultured primary mouse neurons to examine neuronal dysfunction and degeneration observed in patients with TRPV4-related neurodegenerative diseases. Although previous efforts have been made to understand molecular and cellular basis of electrophysiological changes in cell models of TRPV4 mutations, studies in in vivo animal models of neuropathy-causing TRPV4 mutations have yet been examined to assessing biochemical and molecular phenotypes. To assess the consequences of neuropathy-causing mutant TRPV4 expression in neurons in vivo, the authors generated several transgenic overexpression fly lines carrying a mutation in TRPV4 gene causal for several neuropathies (TRPV4-R269C), along with TRPV4 ion-conducting pore mutant and wild-type controls, and performed extensive functional testing, including electrophysiological alterations, including whole-cell patch clamping, calcium imaging, axonal mitochondrial transport, and axonal and dendritic degeneration. The authors report an increase in intracellular Ca2+ in a CaMKII-dependent manner, which impairs axonal mitochondrial transport and axon degeneration. They also confirm these findings in primary mouse neurons. Furthermore, treatment of mutant TRPV4-expressing neurons with selective TRPV4 antagonist or a small peptide CaMKII inhibitor ameliorated mutant TRPV4-mediated neuronal excitability and neurotoxicity. This study importantly advances understanding of molecular genetic pathway of mutant TRPV4-CaMKII-Miro leading to neurotoxicity in neurons in vivo for the first time. Additionally, the paper is well written and tightly argued, references appropriate. My support for publication in Nature Communications is strong.

A minor concern must be addressed. While the authors claim that equivalent titers of lentiviral vectors were transfected into mammalian neurons, there is insufficient assessment of expression levels between WT and mutant TRPV4. The immunoblotting of TRPV4 should include for all genotypes, along with appropriate quantifications to ensure that expression levels are comparable.


Reviewer #2 (Remarks to the Author):

Woolums et al provides strong evidence showing that a disease associated mutation in TRPV4 causes wing phenotype and neurodegeneration when expressed in flies in a gain-of-toxic-function manner. By combining robust fly genetic interactions and pharmacological interventions, the authors show that TRPV4(R269C) causes elevation of intracellular Ca2+ via CamKII, leading to excessive Miro-binding to Ca2+ and the subsequent mitochondrial arrest. Overall the authors have done substantial experiments in multiple systems to support their conclusion and their experiments are well designed and results are convincing. I have one major concern and a technical question as stated below.

Fig.3 demonstrates TRPV4(R269C) increases mean firing rate, which is dependent on basal intracellular Ca2+ levels, because BAPTA application abolishes this phenotype. Consistently, treatment with GSK219, the TRPV4 inhibitor, also abolishes this phenotype. These data clearly suggest that intracellular Ca2+ levels are already elevated by TRPV4(R269C) causing phenotypes in Fig. 3 (also shown in their schematic model). To directly prove that TRPV4(R269C) causes intracellular Ca2+ elevation, in the same experimental settings in Fig. 4, the authors should show basal Ca2+ levels in those mutants; if TRPV4(R269C) mutant has higher Ca2+ concentrations, blocking the channel by the genetic mutation (M680K ) or by the channel inhibitor GSK219, should lower Ca2+ levels. This result would be consistent with the phenotypic rescues by M680K and GSK219 throughout the paper. However, in Fig.4 the authors investigated how different mutations responded to GSK101, an activator of TRPV4. Adding GSK101 to TRPV4(R269C) should allow even more Ca2+ to enter the cell, which was shown to be true by this figure, but this result doesn’t directly validate that TRPV4(R269C) alone already facilitates more Ca2+ entry. In addition, Fig.4 contradicts the results in Fig. 5, which shows that GSK101 cannot further exacerbate the mitochondrial motility phenotype by TRPV4(R269C) – Fig.4 shows GSK101 can further sensitize TRPV4(R269C).

In methods, the authors mentioned that GSK219 was preincubated with larvae for 60 min. Were the larvae dissected (can they survive for 60 min)? Or if not dissected, can the drug penetrate the thick cuticles efficiently? How about the other drugs used in the paper? Were those also preincubated for so long?

**Author rebuttal, first version:**

*Reviewer 1:* *This study importantly advances understanding of molecular genetic pathway of mutant TRPV4-CaMKII-Miro leading to neurotoxicity in neurons in vivo for the first time. Additionally, the paper is well written and tightly argued, references appropriate. My support for publication in Nature Communications is strong.*

**We thank the reviewer for their positive comments.**

*A minor concern must be addressed. While the authors claim that equivalent titers of lentiviral vectors were transfected into mammalian neurons, there is insufficient assessment of expression levels between WT and mutant TRPV4. The immunoblotting of TRPV4 should include for all genotypes, along with appropriate quantifications to ensure that expression levels are comparable*.

**We thank the reviewer for this suggestion. We have now included a quantification of TRPV4 expression in trigeminal neurons transduced with wild type or mutant TRPV4 using native EGFP fluorescence. Importantly, as shown in Supplementary Figure 8c-d, there is no significant difference between wild-type and mutant TRPV4 expression level, and the quantification shown here is from the same set of experiments shown in Figure 5 (same virus and cell culture preparation). We are unable to perform Western analysis on cultured trigeminal neuron preparations due to low level of TRPV4 expression in these neurons. However, we reason that the quantified EGFP fluorescence intensity within neurons is a better experiment since TRPV4:EGFP is also expressed in nonneuronal cells in these cultures. Nonetheless, we did perform Western analysis of HEK293T cells transduced with equivalent titers of lentiviral vectors, and as shown in Supplemental Figure 8a-b, wild-type and mutant TRPV4 express at similar levels.**

*Reviewer 2: Woolums et al provides strong evidence showing that a disease associated mutation in TRPV4 causes wing phenotype and neurodegeneration when expressed in flies in a gain-of-toxic-function manner. By combining robust fly genetic interactions and pharmacological interventions, the authors show that TRPV4(R269C) causes elevation of intracellular Ca2+ via CamKII, leading to excessive Miro-binding to Ca2+ and the subsequent mitochondrial arrest. Overall the authors have done substantial experiments in multiple systems to support their conclusion and their experiments are well designed and results are convincing.*

**We thank the reviewer for their positive comments.**

*1a. Fig.3 demonstrates TRPV4(R269C) increases mean firing rate, which is dependent on basal intracellular Ca2+ levels, because BAPTA application abolishes this phenotype. Consistently, treatment with GSK219, the TRPV4 inhibitor, also abolishes this phenotype. These data clearly suggest that intracellular Ca2+ levels are already elevated by TRPV4(R269C) causing phenotypes in Fig. 3 (also shown in their schematic model). To directly prove that TRPV4(R269C) causes intracellular Ca2+ elevation, in the same experimental settings in Fig. 4, the authors should show basal Ca2+ levels in those mutants; if TRPV4(R269C) mutant has higher Ca2+ concentrations, blocking the channel by the genetic mutation (M680K) or by the channel inhibitor GSK219, should lower Ca2+ levels. This result would be consistent with the phenotypic rescues by M680K and GSK219 throughout the paper. However, in Fig.4 the authors investigated how different mutations responded to GSK101, an activator of TRPV4. Adding GSK101 to TRPV4(R269C) should allow even more Ca2+ to enter the cell, which was shown to be true by this figure, but this result doesn’t directly validate that TRPV4(R269C) alone already facilitates more Ca2+ entry.*

**We thank the reviewer for this suggestion, and we have now analyzed the basal calcium signals in both our *Drosophila* and mammalian neuron models. As the reviewer predicted, basal calcium levels are indeed significantly increased in TRPV4^R269C^ compared with controls in both model systems. As now shown in Supplementary Figure 8e, analysis of basal calcium signals immediately prior to addition of GSK101 in time-lapse images of mouse trigeminal neurons shows a small but statistically significant increase in basal calcium levels in TRPV4^R269C^-expressing neurons compared with TRPV4^WT^-expressing neurons.**

**In live imaging experiments of *Drosophila* larval dendritic arborization (da) neurons, there is a marked increase in the frequency of calcium transients (which we defined as clearly observable, discrete increases in GCaMP5 fluorescence) observed in TRPV4^R269C^-expressing animals that are suppressed when blocking the channel with the M680K mutation (Supplementary Figure 7b and Supplemental Videos 1-4). Importantly, as in the electrophysiology experiments in which R269C causes a marked increase in basal firing rate in CCAP neurons, these imaging experiments are performed in live animals with spontaneous neural activity. We hypothesize that is why the difference in basal calcium signals between R269C and WT is much more striking in flies *in vivo* than in cultured mammalian neurons.**

*1b. In addition, Fig.4 contradicts the results in Fig. 5, which shows that GSK101 cannot further exacerbate the mitochondrial motility phenotype by TRPV4(R269C) – Fig.4 shows GSK101 can further sensitize TRPV4(R269C).*

**We agree with the reviewer that our data show that GSK101 increases intracellular calcium in both *Drosophila* dendritic arbor (da) neurons and primary mouse trigeminal neurons expressing TRPV4^R269C^. The reviewer is also correct to point out that GSK101 does not significantly exacerbate the mitochondrial motility phenotype caused by TRPV4^R269C^ (Figure 7a-b). There are several potential, nonmutually exclusive explanations for this apparent discrepancy, which we have now discussed in our revised manuscript. One possibility is that TRPV4^R269C^ alone already maximally inhibits axonal transport, potentially by saturable binding of calcium to Miro, and therefore further increasing calcium with GSK101 does not further inhibit mitochondrial transport. Second, mitochondrial motility measurements are likely not as sensitive to changes in calcium as are GCaMP and Fura-2 imaging, possibly due to Miro-independent mitochondrial trafficking. Indeed, as shown in Figure 7b, there is a slight enhancement of the mitochondrial motility phenotype in TRPV4^R269C^ with GSK101, though it does not reach statistical significance. Third, it is also likely that there are compartmental differences in calcium levels that might explain differential effects of GSK101 in the different assays. Our GCaMP and Fura-2 imaging experiments measure calcium changes in the cell soma, whereas our mitochondrial motility experiments assess changes in axons. Thus, there are several potential reasons that GSK101 may not further enhance mitochondrial motility phenotypes in TRPV4^R269C^ expressing neurons.**

*2. In methods, the authors mentioned that GSK219 was preincubated with larvae for 60 min. Were the larvae dissected (can they survive for 60 min)? Or if not dissected, can the drug penetrate the thick cuticles efficiently? How about the other drugs used in the paper? Were those also preincubated for so long?*

**We have clarified the methods for pharmacological experiments used in larval experiments in the online methods section of the manuscript. GSK101 was added directly to the media of dissected third-instar larval fillets during GCaMP imaging experiments. For mitochondrial motility experiments with GSK219, larvae were raised on food with 10 μM GSK219, and 10 μM GSK219 was also added to the recording media due to the observed insensitivity of the mutant channel to GSK219 in electrophysiology experiments (Fig. 4c). For mitochondrial motility experiments with GSK101, drug was added to the recording media 1-2 min prior to recording. The 60 min to which the reviewer refers do not pertain to larval fillets experiments, but rather only to the electrophysiological recordings on N_CCAP_. In these experiments** **during the preincubation period, 95% O_2_ and 5% CO_2_ pre-bubbled *Drosophila* physiological saline solution containing the drug was continuously perfused by Peristaltic pump (MINIPULS 3, Gilson) with flow rate of 2-3 ml/min. Using similar preparations, others have observed electrophysiological viability of *Drosophila* brains for as long as 2 hours post-dissection (Ueno et al. J Physiol. 2013)**

**Reviewer comments, second version:**

REVIEWERS' COMMENTS:

Reviewer #1 (Remarks to the Author):

This revised manuscript well addresses all concerns and issues raised by me and other reviewer. The authors have made this a very good manuscript and have been well responsive to our criticisms. I believe this work will provide valuable insights into molecular genetic basis of TRPV4 signaling pathway in neurotoxicity.

Janghoo Lim


Reviewer #2 (Remarks to the Author):

The authors have sufficiently addressed my concerns.

**Author rebuttal, second version:**

**Reviewer comments, third version:**

**Author rebuttal, third version:**
